# *Staphylococcus aureus* Uses the Bacilliredoxin (BrxAB)/Bacillithiol Disulfide Reductase (YpdA) Redox Pathway to Defend Against Oxidative Stress Under Infections

**DOI:** 10.3389/fmicb.2019.01355

**Published:** 2019-06-18

**Authors:** Nico Linzner, Vu Van Loi, Verena Nadin Fritsch, Quach Ngoc Tung, Saskia Stenzel, Markus Wirtz, Rüdiger Hell, Chris J. Hamilton, Karsten Tedin, Marcus Fulde, Haike Antelmann

**Affiliations:** ^1^Institute for Biology – Microbiology, Freie Universität Berlin, Berlin, Germany; ^2^Plant Molecular Biology, Centre for Organismal Studies Heidelberg, Heidelberg University, Heidelberg, Germany; ^3^School of Pharmacy, University of East Anglia, Norwich, United Kingdom; ^4^Institute of Microbiology and Epizootics, Centre for Infection Medicine, Freie Universität Berlin, Berlin, Germany

**Keywords:** *Staphylococcus aureus*, oxidative stress, bacillithiol, bacilliredoxin, bacillithiol disulfide reductase, YpdA, roGFP2

## Abstract

*Staphylococcus aureus* is a major human pathogen and has to cope with reactive oxygen and chlorine species (ROS, RCS) during infections. The low molecular weight thiol bacillithiol (BSH) is an important defense mechanism of *S. aureus* for detoxification of ROS and HOCl stress to maintain the reduced state of the cytoplasm. Under HOCl stress, BSH forms mixed disulfides with proteins, termed as *S*-bacillithiolations, which are reduced by bacilliredoxins (BrxA and BrxB). The NADPH-dependent flavin disulfide reductase YpdA is phylogenetically associated with the BSH synthesis and BrxA/B enzymes and was recently suggested to function as BSSB reductase (Mikheyeva et al., 2019). Here, we investigated the role of the complete bacilliredoxin BrxAB/BSH/YpdA pathway in *S. aureus* COL under oxidative stress and macrophage infection conditions *in vivo* and in biochemical assays *in vitro*. Using HPLC thiol metabolomics, a strongly enhanced BSSB level and a decreased BSH/BSSB ratio were measured in the *S. aureus* COL Δ*ypdA* deletion mutant under control and NaOCl stress. Monitoring the oxidation degree (OxD) of the Brx-roGFP2 biosensor revealed that YpdA is required for regeneration of the reduced BSH redox potential (*E*_BSH_) upon recovery from oxidative stress. In addition, the Δ*ypdA* mutant was impaired in H_2_O_2_ detoxification as measured with the novel H_2_O_2_-specific Tpx-roGFP2 biosensor. Phenotype analyses further showed that BrxA and YpdA are required for survival under NaOCl and H_2_O_2_ stress *in vitro* and inside murine J-774A.1 macrophages in infection assays *in vivo*. Finally, NADPH-coupled electron transfer assays provide evidence for the function of YpdA in BSSB reduction, which depends on the conserved Cys14 residue. YpdA acts together with BrxA and BSH in de-bacillithiolation of *S*-bacillithiolated GapDH. In conclusion, our results point to a major role of the BrxA/BSH/YpdA pathway in BSH redox homeostasis in *S. aureus* during recovery from oxidative stress and under infections.

## Introduction

*Staphylococcus aureus* is an important human pathogen, which can cause many diseases, ranging from local soft-tissue and wound infections to life-threatening systemic and chronic infections, such as endocarditis, septicaemia, bacteraemia, pneumonia or osteomyelitis (Archer, 1998; Lowy, 1998; Boucher and Corey, 2008). Due to the prevalence of methicillin-resistant *S. aureus* isolates, which are often resistant to multiple antibiotics, treatment options are limited to combat *S. aureus* infections (Livermore, 2000). Therefore, the “European Center of Disease Prevention and Control” has classified *S. aureus* as one out of six ESKAPE pathogens which are the leading causes of nosocomial infections worldwide (Pendleton et al., 2013). During infections, activated macrophages and neutrophils produce reactive oxygen and chlorine species (ROS, RCS) in large quantities, including H_2_O_2_ and HOCl with the aim to kill invading pathogens (Winterbourn and Kettle, 2013; Hillion and Antelmann, 2015; Beavers and Skaar, 2016; Winterbourn et al., 2016).

**FIGURE 1:**
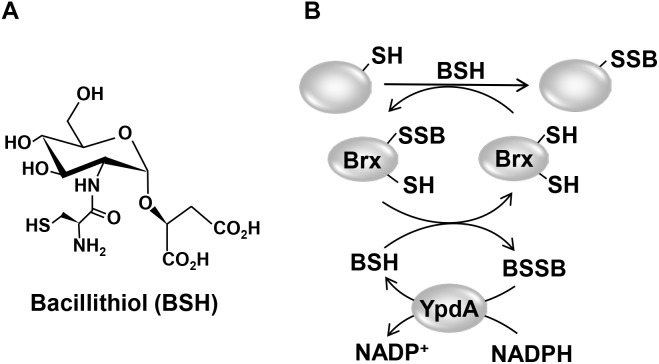
Structure of the LMW thiol bacillithiol (BSH) **(A)** and mechanism of the bacilliredoxin (Brx)/BSH/YpdA de-bacillithiolation pathway **(B)**. **(A)** Bacillithiol is composed of glucosamine, malate, and cysteine. **(B)** Under HOCl stress, BSH leads to *S*-bacillithiolation of proteins which are reduced by bacilliredoxins (BrxA/B), resulting in the transfer of BSH to the Brx active site (Brx-SSB). BSH functions in Brx-SSB reduction to restore Brx activity, leading to BSSB formation. The BSSB reductase YpdA (SACOL1520) regenerates BSH on expense of NADPH.

Low molecular weight thiols play important roles in the defense against ROS and HOCl in bacterial pathogens and are required for survival, host colonization, and pathogenicity (Loi et al., 2015; Tung et al., 2018). Gram-negative bacteria produce GSH as major LMW thiol, which is absent in most Gram-positive bacteria (Fahey, 2013). Instead, many firmicutes utilize BSH as alternative LMW thiol (Figure 1A), which is essential for virulence of *S. aureus* in macrophage infection assays (Newton et al., 2012; Pöther et al., 2013; Posada et al., 2014; Chandrangsu et al., 2018). A recent study identified a BSH derivative with an *N*-methylated cysteine as *N*-methyl-BSH in anaerobic phototrophic *Chlorobiaceae*, suggesting that BSH derivatives are more widely distributed and not restricted to Gram-positive firmicutes (Hiras et al., 2018). In *S. aureus* and *Bacillus subtilis*, BSH was characterized as cofactor of thiol-*S*-transferases (e.g., FosB), glyoxalases, peroxidases, and other redox enzymes that are involved in detoxification of ROS, HOCl, methylglyoxal, toxins, and antibiotics (Chandrangsu et al., 2018). In addition, BSH participates in post-translational thiol-modifications under HOCl stress by formation of BSH mixed protein disulfides, termed as protein *S*-bacillithiolations (Chi et al., 2011, 2013; Imber et al., 2018a,c).

Protein *S*-bacillithiolation functions in thiol-protection and redox regulation of redox-sensing regulators, metabolic enzymes and antioxidant enzymes (Chi et al., 2011, 2013; Loi et al., 2015; Imber et al., 2018a,b,c). In *S. aureus*, the glycolytic glyceraldehyde-3-phosphate dehydrogenase (GapDH) and the aldehyde dehydrogenase AldA were identified as most abundant *S*-bacillithiolated proteins that are inactivated under HOCl stress (Imber et al., 2018a,b). In *B. subtilis*, the methionine synthase MetE and the OhrR repressor are *S*-bacillithiolated under HOCl stress leading to methionine auxotrophy and derepression of the OhrR-controlled *ohrA* peroxiredoxin gene, respectively (Fuangthong et al., 2001; Lee et al., 2007; Chi et al., 2011).

Reduction of *S*-bacillithiolated OhrR, MetE, and GapDH proteins is catalyzed by the bacilliredoxins (BrxA/B) in *B. subtilis* and *S. aureus in vitro* (Gaballa et al., 2014; Chandrangsu et al., 2018). BrxA (YphP) and BrxB (YqiW) are paralogous thioredoxin-fold proteins of the UPF0403 family with an unusual CGC active site that are conserved in BSH-producing firmicutes (Supplementary Figure S1). Upon de-bacillithiolation, the BSH moiety is transferred to the Brx active site, resulting in BrxA-SSB formation (Figure 1B). However, the Brx associated thiol-disulfide reductase involved in regeneration of Brx activity is not known. In GSH-producing bacteria, Grx catalyze the reduction of *S*-glutathionylated proteins, which requires GSH for regeneration of Grx, resulting in GSSG formation (Lillig et al., 2008; Allen and Mieyal, 2012). The regeneration of GSH is catalyzed by the flavoenzyme Gor, which belongs to the pyridine nucleotide disulfide reductases and recycles GSSG on expense of NADPH (Argyrou and Blanchard, 2004; Deponte, 2013).

Phylogenomic profiling of protein interaction networks using EMBL STRING search has suggested the flavoenzyme YpdA (SACOL1520) as putative NADPH-dependent BSSB reductase (Supplementary Figure S1), since YpdA co-occurs together with BrxA/B and the BSH biosynthesis enzymes (BshA/B/C) only in BSH-producing bacteria, such as *B. subtilis* and *S. aureus* (Supplementary Figure S2; Gaballa et al., 2010). While our work was in progress, a recent study provides first evidence for the function of YpdA as putative BSSB reductase in *S. aureus in vivo* since an increased BSSB level and a decreased BSH/BSSB ratio was measured in the Δ*ypdA* mutant under control and H_2_O_2_ stress conditions (Mikheyeva et al., 2019). YpdA overproduction was shown to increase the BSH level and contributes to oxidative stress resistance, fitness, and virulence of *S. aureus* (Mikheyeva et al., 2019). However, biochemical evidence for the function of YpdA as BSSB reductase and the association of YpdA to the BrxA/B enzymes have not been demonstrated in *B. subtilis* or *S. aureus*.

In this work, we aimed to investigate the role of the complete BrxAB/BSH/YpdA pathway in *S. aureus in vivo* and *in vitro*. We used phenotype and biochemical analyses, HPLC metabolomics and redox biosensor measurements to study the physiological role of the Brx/BSH/YpdA redox pathway in *S. aureus* under oxidative stress and macrophage infection assays. Our data point to important roles of both BrxA and YpdA in the oxidative stress defense for regeneration of reduced *E*_BSH_ and de-bacillithiolation upon recovery from oxidative stress. Biochemical assays further provide evidence for the function of YpdA as BSSB reductase *in vitro*, which acts in the BrxA/BSH/YpdA electron pathway in de-bacillithiolation of GapDH-SSB.

## Materials and Methods

### Bacterial Strains, Growth, and Survival Assays

Bacterial strains, plasmids and primers used in this study are listed in Supplementary Tables S1, S2, S3. For cloning and genetic manipulation, *Escherichia coli* was cultivated in LB medium. For stress experiments, *S. aureus* COL wild type and mutant strains were cultivated in LB, RPMI, or Belitsky minimal medium and exposed to the different compounds during the exponential growth as described previously (Loi et al., 2017, 2018b). NaOCl, methylglyoxal, diamide, methylhydroquinone, DTT, cumene hydroperoxide (80% w/v), H_2_O_2_ (35% w/v), and monobromobimane were purchased from Sigma Aldrich.

### Cloning, Expression, and Purification of His-Tagged Brx-roGFP2, Tpx-roGFP2, GapDH, BrxA, YpdA, and YpdAC14A Proteins in *E. coli*

Construction of plasmids pET11b-*brx-roGFP2* for expression of the Brx-roGFP2 biosensor was described previously (Loi et al., 2017). The pET11b-derived plasmids for overexpression of the His-tagged GapDH and BrxA (SACOL1321) proteins were generated previously (Imber et al., 2018a). The plasmid pET11b-*brx-roGFP2* was used as a template for construction of the Tpx-roGFP2 biosensor to replace *brx* by the *tpx* gene of *S. aureus*. The *tpx* gene (*SACOL1762*) was PCR-amplified from chromosomal DNA of *S. aureus* COL using primers pET-tpx-for-NheI and pET-tpx-rev-SpeI (Supplementary Table S3), digested with *Nhe*I and *Bam*HI and cloned into plasmid pET11b-*brx-roGFP2* to generate pET11b-*tpx-roGFP2*. To construct plasmids pET11b-*ypdA* or pET11b-*ypdAC14A*, the *ypdA* gene (*SACOL1520*) was PCR-amplified from chromosomal DNA of *S. aureus* COL with pET-ypdA-for-NdeI or pET-ypdAC14A-for-NdeI as forward primers and pET-ypdA-rev-BamHI as reverse primer (Supplementary Table S3), digested with *Nde*I and *Bam*HI and inserted into plasmid pET11b (Novagen). For expression of His-tagged proteins (GapDH, BrxA, YpdA, YpdAC14A, Tpx-roGFP2), *E. coli* BL21(DE3) *plys*S carrying plasmids pET11b-*gap*, pET11b-*brxA*, pET11b-*ypdA*, pET11b-*ypdAC14A* and pET11b-*tpx-roGFP2* was cultivated in 1 l LB medium until an OD_600_ of 0.8 followed by addition of 1 mM IPTG (isopropyl-β-D-thiogalactopyranoside) for 16 h at 25°C. His_6_-tagged GapDH, BrxA, YpdA, YpdAC14A, and Tpx-roGFP2 proteins were purified using His Trap^TM^ HP Ni-NTA columns (5 ml; GE Healthcare, Chalfont St Giles, United Kingdom) and the ÄKTA purifier liquid chromatography system (GE Healthcare) as described (Loi et al., 2018b).

### Construction of *S. aureus* COL Δ*ypdA*,Δ*brxAB* and Δ*brxAB*Δ*ypdA* Clean Deletion Mutants and Complemented Mutant Strains

*Staphylococcus aureus* COL Δ*ypdA* (*SACOL1520*), Δ*brxA* (*SACOL1464*), and Δ*brxB* (*SACOL1558*) single deletion mutants as well as the Δ*brxAB* double and Δ*brxAB*Δ*ypdA* triple mutants were constructed using pMAD as described (Arnaud et al., 2004; Loi et al., 2018b). Briefly, the 500 bp up- and downstream regions of *ypdA, brxA*, and *brxB* were amplified using gene-specific primers (Supplementary Table S3), fused by overlap extension PCR and ligated into the *Bgl*II and *Sal*I sites of plasmid pMAD. The pMAD constructs were electroporated into *S. aureus* RN4220 and further transduced into *S. aureus* COL using phage 81 (Rosenblum and Tyrone, 1964). The clean marker-less deletions of *ypdA, brxA*, or *brxB* were selected after plasmid excision as described (Loi et al., 2018b). All mutants were clean deletions of internal gene regions with no genetic changes in the up- and downstream encoding genes. The deletions of the internal gene regions were verified by PCR and DNA sequencing. The Δ*brxAB* and Δ*brxAB*Δ*ypdA* double and triple mutants were obtained by transduction and excision of pMAD-Δ*brxB* into the Δ*brxA* mutant, leading to the Δ*brxAB* deletion and of plasmid pMAD-Δ*ypdA* into the Δ*brxAB* mutant, resulting in the Δ*brxAB*Δ*ypdA* knockout. For construction of *ypdA, brxA*, and *brxB* complemented strains, the xylose-inducible ectopic *E. coli/S. aureus* shuttle vector pRB473 was applied (Brückner et al., 1993). Primers pRB-ypdA, pRB-brxA, and pRB-brxB (Supplementary Table S3) were used for amplification of the genes, which were cloned into pRB473 after digestion with *Bam*HI and *Kpn*I to generate plasmids pRB473-*ypdA*, pRB473-*brxA*, and pRB473-*brxB*, respectively. The pRB473 constructs were confirmed by PCR and DNA sequencing and transduced into the Δ*ypdA* and Δ*brxAB* deletion mutants as described (Loi et al., 2017).

### Construction of Tpx-roGFP2 and Brx-roGFP2 Biosensor Fusions in *S. aureus* COL

The *tpx-roGFP2* fusion was amplified from plasmid pET11b-*tpx-roGFP2* with primers pRB-tpx-roGFP2-for-BamHI and pRB-tpx-roGFP2-rev-SacI and digested with *Bam*HI and *Sac*I (Supplementary Table S3). The PCR product was cloned into pRB473 generating plasmid pRB473-*tpx-roGFP2*, which was confirmed by DNA sequencing. The biosensor plasmids pRB473-*tpx-roGFP2* and pRB473-*brx-roGFP2* were electroporated into *S. aureus* RN4220 and further transferred to the *S. aureus* COL Δ*ypdA*, Δ*brxAB* and Δ*brxAB*Δ*ypdA* mutants by phage transduction as described (Loi et al., 2017).

### Northern Blot Experiments

Northern blot analyses were performed using RNA isolated from *S. aureus* COL before and 15 min after exposure to 0.5 mM methylglyoxal, 0.75 mM formaldehyde, 1 mM NaOCl, 10 mM H_2_O_2_, 2 mM diamide, and 45 μM methylhydroquinone as described (Wetzstein et al., 1992). Hybridizations were conducted using digoxigenin-labeled antisense RNA probes for *ypdA, brxA*, and *brxB* that were synthesized *in vitro* using T7 RNA polymerase and primers ypdA-NB-for/rev, brxA-NB-for/rev, or brxB-NB-for/rev (Supplementary Table S3) as in previous studies (Tam le et al., 2006).

### HPLC Thiol Metabolomics for Quantification of LMW Thiols and Disulfides

For preparation of thiol metabolomics samples, *S. aureus* COL WT, Δ*ypdA* and Δ*brxAB* mutants as well as the *ypdA* complemented strains were grown in RPMI medium to an OD_500_ of 0.9 and exposed to 2 mM NaOCl stress for 30 min. The intracellular amounts of reduced and oxidized LMW thiols and disulfides (BSH, BSSB, cysteine and cystine) were extracted from the *S. aureus* cells, labeled with monobromobimane and measured by HPLC thiol metabolomics as described (Chi et al., 2013).

### Western Blot Analysis

*Staphylococcus aureus* strains were grown in LB until an OD_540_ of 2, transferred to Belitsky minimal medium and treated with 100 μM NaOCl for 60 and 90 min. Cytoplasmic proteins were prepared and subjected to non-reducing BSH-specific Western blot analysis using the polyclonal rabbit anti-BSH antiserum as described previously (Chi et al., 2013). The de-bacillithiolation reactions with purified GapDH-SSB and the BrxA/BSH/YpdA/NADPH pathway were also subjected to non-reducing BSH-specific Western blots.

### Brx-roGFP2 and Tpx-roGFP2 Biosensor Measurements

*Staphylococcus aureus* COL, Δ*ypdA* and Δ*brxAB* mutant strains expressing the Brx-roGFP2 and Tpx-roGFP2 biosensor plasmids were grown in LB and used for measurements of the biosensor oxidation degree (OxD) along the growth curves and after injection of the oxidants H_2_O_2_ and NaOCl as described previously (Loi et al., 2017). The fully reduced and oxidized control samples of Tpx-roGFP2 expression strains were treated with 15 mM DTT and 20 mM cumene hydroperoxide, respectively. The Brx-roGFP2 and Tpx-roGFP2 biosensor fluorescence emission was measured at 510 nm after excitation at 405 and 488 nm using the CLARIOstar microplate reader (BMG Labtech). The OxD of the Brx-roGFP2 and Tpx-roGFP2 biosensors was determined for each sample and normalized to fully reduced and oxidized controls as described (Loi et al., 2017) according to the Eq. (1):

(1) $$O\times D=\frac{I405_{\mathrm{sample}}\times I488_{\mathrm{red}}-I405_{\mathrm{red}}\times I488_{\mathrm{sample}}}{I405_{\mathrm{sample}}\times I488_{\mathrm{red}}-I405_{\mathrm{sample}}\times I488_{\mathrm{ox}}+I405_{\mathrm{ox}}\times I488_{\mathrm{sample}}-I405_{\mathrm{red}}\times I488_{\mathrm{sample}}}$$

The values of *I*405_sample_ and *I*488_sample_ are the observed fluorescence excitation intensities at 405 and 488 nm, respectively. The values of *I*405_red_, *I*488_red_, *I*405_ox_, and *I*488_ox_ represent the fluorescence intensities of fully reduced and oxidized controls, respectively.

Based on the OxD values and the previously determined $E_{\mathrm{roGFP}2}^{0^{\prime }}=-280\;\mathrm{mV}$ (Dooley et al., 2004), the BSH redox potential (*E*_BSH_) can be calculated using to the Nernst equation (2):

(2) $$E_{\mathrm{BSH}}=E_{\mathrm{roGFP}2}=E_{\mathrm{roGFP}2}^{0^{\prime }}-\left(\frac{\mathrm{RT}}{2F}\right)\times \mathrm{In}\;\left(\frac{1-O\times D}{O\times D}\right)$$

### Biochemical Assays for NADPH-Dependent BSSB Reduction by YpdA and De-Bacillithiolation of GapDH-SSB Using the BrxA/BSH/YpdA Electron Pathway *in vitro*

Before the activity assays, the purified BrxA, YpdA, and YpdAC14A proteins were prereduced with 10 mM DTT followed by DTT removal with Micro Biospin 6 columns (Biorad). For the biochemical activity assays of the specific BSSB reductase activity, 12.5 μM of purified YpdA and YpdAC14A proteins were incubated with 40 μM BSSB, 40 μM GSSG, or 40 μM coenzyme A disulfide and 500 μM NADPH in 20 mM Tris, 1.25 mM EDTA, pH 8.0. NADPH consumption of YpdA and YpdAC14A was measured immediately after the start of the reaction as absorbance change at 340 nm using the Clariostar microplate reader. The NADPH-dependent BrxA/BSH/YpdA electron pathway was reconstituted *in vitro* for de-bacillithiolation of GapDH-SSB. About 60 μM of purified GapDH was *S*-bacillithiolated with 600 μM BSH in the presence of 6 mM H_2_O_2_ for 5 min. Excess of BSH and H_2_O_2_ were removed with Micro Biospin 6 columns, which were equilibrated with 20 mM Tris, 1.25 mM EDTA, pH 8.0. Before starting the de-bacillithiolation assay using the BrxA/BSH/YpdA electron pathway, 2.5 μM GapDH-SSB was incubated with 12.5 μM BrxA, 40 μM BSH, and 500 μM NADPH in 20 mM Tris, 1.25 mM EDTA, pH 8.0 at room temperature for 30 min. Next, 12.5 μM YpdA or YpdAC14A proteins were added to the reaction mix at 30°C for 8 min and NADPH consumption was measured at 340 mm. The biochemical activity assays were performed in four replicate experiments.

### Infection Assays With Murine Macrophage Cell Line J-774A.1

The murine cell line J774A.1 was cultivated in Iscove’s modified Dulbecco MEM medium (Biochrom) with 10% heat inactivated fetal bovine serum (FBS) and used for *S. aureus* infection assays as described (Loi et al., 2018b). Macrophages were infected with *S. aureus* cells at a multiplicity of infection (MOI) of 1:25. One hour after infection, the cell culture medium was replaced and 150 μg/ml gentamycin was added for 1 h to kill extracellular bacteria and to stop the uptake of *S. aureus*. The *S. aureus* cells were harvested at 2, 4, and 24 h post infection. To determine the percentage of surviving *S. aureus* cells, infected macrophages were lysed with 0.1% Triton X-100 and the supernatant of internalized bacteria was plated on brain heart infusion (BHI) agar plates. The CFUs were counted after incubation for 24–36 h at 37°C (Loi et al., 2018b).

### Statistical Analyses

Statistical analysis of growth and survival assays was performed using the Student’s unpaired two-tailed *t*-test by the graph prism software. The statistics of the J-774.1 macrophage infection assays was calculated using the one-way ANOVA and Tukey’s multiple comparisons *post hoc* test by the graph prism software. The results of the statistical tests are included in the figure legends.

## Results

### Transcription of *ypdA, brxA*, and *brxB* Is Induced Under Disulfide Stress by Diamide and NaOCl in *S. aureus* COL

**FIGURE 2:**
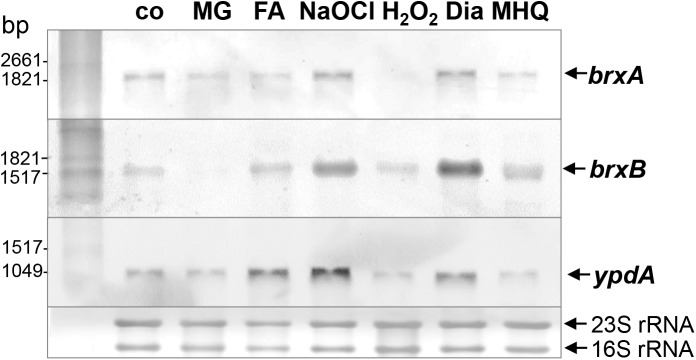
Transcription of *brxA, brxB*, and *ypdA* is induced by disulfide stress in *S. aureus*. Northern blot analysis was used to analyze transcription of *brxA, brxB*, and *ypdA* in *S. aureus* COL wild type before (co) and 15 min after exposure to 0.5 mM methylglyoxal (MG), 0.75 mM formaldehyde (FA), 1 mM NaOCl, 10 mM H_2_O_2_, 2 mM diamide (Dia), and 45 μM methylhydroquinone (MHQ) stress at an OD_500_ of 0.5. The arrows point toward the transcript sizes of the *brxA, brxB*, and *ypdA* specific genes and operons. The methylene blue-stained bands of the 16S and 23S rRNAs are shown as RNA loading control at the bottom.

The bacilliredoxins BrxA (SACOL1464) and BrxB (SACOL1558) of *S. aureus* share an unusual CGC active site and are highly conserved in BSH-producing firmicutes (Supplementary Figure S1; Gaballa et al., 2014). The pyridine nucleotide disulfide oxidoreductase YpdA (SACOL1520) belongs to the FAD/NAD(P)-binding domain superfamily (IPR036188) and was annotated as putative BSSB reductase due to its phylogenetic co-occurence with the BSH biosynthesis enzymes and BrxA/B in BSH-producing firmicutes (Supplementary Figure S2; Gaballa et al., 2010). We used Northern blot analysis to investigate whether transcription of *brxA, brxB*, and *ypdA* is co-regulated and up-regulated under thiol-specific stress conditions, such as 0.5 mM methylglyoxal, 0.75 mM formaldehyde, 1 mM NaOCl, 10 mM H_2_O_2_, 2 mM diamide and 45 μM methylhydroquinone (Figure 2). The *brxA* gene is co-transcribed with *SACOL1465-66-67* in a 2 kb operon and *brxB* is located in the 1.6 kb *SACOL1557-brxB-SACOL1559* operon. The genes co-transcribed together with *brxA* and *brxB* encode proteins of unknown functions. The Northern blot results revealed significant basal transcription of the *brxA, brxB*, and *ypdA* genes and operons in the control, and strong induction under disulfide stress provoked by NaOCl and diamide. Of note, the *brxB* operon was stronger induced under disulfide stress compared to the *brxA* operon (Figure 2). No up-regulation of the *brxA, brxB*, and *ypdA* specific mRNAs was detected upon H_2_O_2_, aldehyde and quinone stress. The co-regulation of BrxA/B and YpdA under disulfide stress suggests that they act in the same pathway to regenerate *S*-bacillithiolated proteins under NaOCl stress upon recovery from oxidative stress.

### The BSSB Level Is Significantly Increased and the BSH/BSSB Ratio Is Decreased in the *S. aureus* Δ*ypdA* Mutant

**FIGURE 3:**
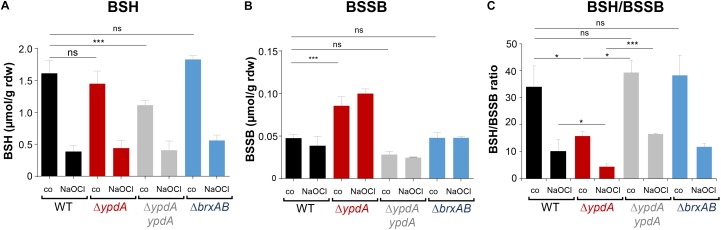
The BSSB level is strongly increased and the BSH/BSSB redox ratio is decreased in the *S. aureus* COL Δ*ypdA* mutant under control and NaOCl stress. The levels of BSH **(A)**, BSSB **(B)** and the BSH/BSSB redox ratio **(C)** were determined in *S. aureus* COL wild type (WT), the Δ*ypdA* and Δ*brxAB* mutants as well as in the *ypdA* complemented strain before (co) and 30 min after treatment with 2 mM NaOCl stress. LMW thiols and disulfides were labeled with monobromobimane and measured using HPLC-thiol-metabolomics. Mean values and standard deviations (SD) of four biological replicates are shown. ^ns^*p* > 0.05; ^∗^*p* ≤ 0.01, and ^∗∗∗^*p* ≤ 0.001.

To investigate the physiological role of BrxA/B and YpdA under oxidative stress and in BSH redox homeostasis, we constructed Δ*brxAB* and Δ*ypdA* deletion mutants. Using HPLC thiol metabolomics, the intracellular levels of BSH and BSSB were determined in the Δ*brxAB* and Δ*ypdA* mutants under control and NaOCl stress after monobromobimane derivatisation of LMW thiols and disulfides. In the *S. aureus* COL wild type, a BSH level of 1.6–1.9 μmol/g rdw was determined, which was not significantly different in the Δ*ypdA* and Δ*brxAB* mutants (Figure 3A). Exposure of *S. aureus* to 2 mM NaOCl stress caused a five to sixfold decreased intracellular BSH level in the wild type, Δ*ypdA* and Δ*brxAB* mutants (Figure 3A). The level of BSSB was similar in control and NaOCl-treated cells of the wild type and Δ*brxAB* mutant (∼0.05 μmol/g rdw) (Figure 3B). Most interestingly, the Δ*ypdA* mutant showed a significantly twofold increased BSSB level under control and NaOCl stress compared to the wild type (Figure 3B), confirming previous data (Mikheyeva et al., 2019). Thus, the BSH/BSSB ratio is ∼2–3-fold decreased in the Δ*ypdA* mutant under control and NaOCl relative to the parent (Figure 3C). The increased BSSB levels and the decreased BSH/BSSB redox ratio in the Δ*ypdA* mutant could be restored to wild type levels in the *ypdA* complemented strain. In addition, a significantly 1.5-fold increased cysteine level was measured in the Δ*ypdA* mutant under NaOCl stress, but no changes in the level of cystine (Supplementary Figures S3A–C). The cysteine levels could be also restored to wild type level in the *ypdA* complemented strain. These results indicate that YpdA is important to maintain the reduced level of BSH under control and NaOCl stress, supporting previous results (Mikheyeva et al., 2019), while the bacilliredoxins BrxA/B are dispensible for the cellular BSH/BSSB redox balance during the growth and under oxidative stress in *S. aureus*.

### The *S. aureus*Δ*ypdA* Mutant Is Impaired to Regenerate the Reduced BSH Redox Potential and to Detoxify H_2_O_2_ Under Oxidative Stress

**FIGURE 4:**
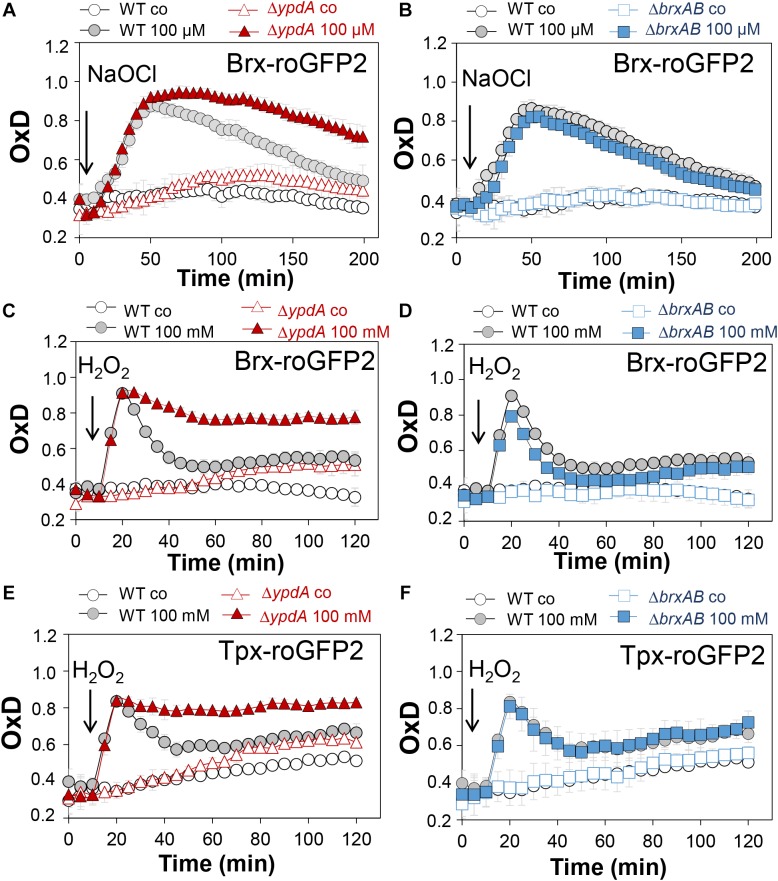
Brx-roGFP2 and Tpx-roGFP2 biosensors measurements of the OxD indicate that the *S. aureus*Δ*ypdA* mutant is impaired to regenerate the reduced state of *E*_BSH_ and to detoxify H_2_O_2_ during recovery from oxidative stress. **(A–D)** Response of the Brx-roGFP2 biosensor to 100 μM NaOCl and 100 mM H_2_O_2_ stress in *S. aureus* COL WT, the Δ*ypdA* **(A,C)** and Δ*brxAB* **(B,D)** mutants. **(E,F)** Response of the Tpx-roGFP2 biosensor under 100 mM H_2_O_2_ stress in the *S. aureus* COL WT, the Δ*ypdA* and Δ*brxAB* mutants. The Brx-roGFP2 biosensor responses are shown as OxD values and the corresponding *E*_BSH_ changes were calculated using the Nernst equation and presented in Supplementary Figure S5 and Supplementary Tables S5, S6. Mean values and SD of three biological replicates are shown.

Next, we applied the Brx-roGFP2 biosensor to monitor the changes of its OxD in *S. aureus* COL wild type, the Δ*ypdA* and Δ*brxAB* mutants during the growth and under oxidative stress (Loi et al., 2017). Using the Nernst equation the OxD values were used to calculate the changes in the BSH redox potential (*E*_BSH_) in wild type and mutant strains (see section “Materials and Methods” for details). Measurements of the Brx-roGFP2 OxD in LB medium along the growth did not reveal notable differences in the basal level of *E*_BSH_ between wild type, Δ*ypdA* and Δ*brxAB* mutant strains (Supplementary Figures S4A,B, S5A,B and Supplementary Table S4). The basal level of *E*_BSH_ varied from -282 to -295 mV in the wild type and from -286 to -299 mV in the Δ*ypdA* and Δ*brxAB* mutants in different growth phases (Supplementary Figures S5A,B and Supplementary Table S4). Thus, we monitored the biosensor OxD and calculated the *E*_BSH_ changes in Δ*ypdA* and Δ*brxAB* mutants after exposure to sub-lethal doses of 100 μM NaOCl and 100 mM H_2_O_2_ to identify functions for BrxAB or YpdA under oxidative stress. The Brx-roGFP2 biosensor was strongly oxidized under NaOCl and H_2_O_2_ stress in the wild type, the Δ*ypdA* and Δ*brxAB* mutants (Figure 4A–D). The calculated *E*_BSH_ increased upon NaOCl stress from -286 to -254 mV in the wild type, from -285 to -247 mV in the Δ*ypdA* mutant and from -288 to -259 mV in the Δ*brxAB* mutant (Supplementary Figures S5C,D and Supplementary Table S5). This indicates a stronger increase of *E*_BSH_ by NaOCl stress in the Δ*ypdA* mutant compared to the wild type. Regeneration of the reduced basal level *E*_BSH_ occurred already after 2 h reaching values of -269 mV in the wild type and -274 mV in the Δ*brxAB* mutant (Figure 4B, Supplementary Figure S5D, and Supplementary Table S5). However, the Δ*ypdA* mutant was significantly impaired to recover the reduced state and *E*_BSH_ values remained high with -252 mV after 2 h of NaOCl stress (Figure 4A, Supplementary Figure S5C, and Supplementary Table S5). Of note, the defect of the Δ*ypdA* mutant to restore the reduced state of *E*_BSH_ was reproducible with both oxidants, H_2_O_2_ and NaOCl (Figure 4A,C, Supplementary Figures S5C,E, and Supplementary Table S6). While recovery of reduced *E*_BSH_ after H_2_O_2_ stress was fast in the wild type and Δ*brxAB* mutant reaching *E*_BSH_ values of -280 and -283 mV already after 60 min, the Δ*ypdA* mutant was still oxidized after 2 h with high *E*_BSH_ values of -264 mV (Supplementary Figures S5E,F and Supplementary Table S6). These Brx-roGFP2 measurements document the important role of YpdA to reduce BSSB and to regenerate the reduced *E*_BSH_ during the recovery phase of cells from oxidative stress.

We further hypothesized that the Δ*ypdA* mutant is defective in H_2_O_2_ detoxification due to its increased BSSB levels. To analyse the kinetics of H_2_O_2_ detoxification in the Δ*ypdA* mutant, we constructed a genetically encoded H_2_O_2_-specific Tpx-roGFP2 biosensor. First, we verified that Tpx-roGFP2 showed the same ratiometric changes of the excitation spectrum in the fully reduced and oxidized state *in vitro* and *in vivo* as previously measured for Brx-roGFP2 (Supplementary Figures S6A,B). Tpx-roGFP2 was shown to respond strongly to low levels of 0.5–1 μM H_2_O_2_ *in vitro* and was fully oxidized with 100 mM H_2_O_2_ inside *S. aureus* COL wild type cells indicating the utility of the probe to measure H_2_O_2_ detoxification kinetics in *S. aureus* (Supplementary Figures S6C,D). Measurements of Tpx-roGFP2 oxidation along the growth in LB medium revealed a similar high OxD of ∼0.5–0.6 in the wild type, Δ*brxAB* and Δ*ypdA* mutant strains (Supplementary Figures S4C,D). The absence of BrxA/B or YpdA did not affect the biosensor OxD under non-stress conditions, which further provides evidence for roles under oxidative stress. Thus, we monitored the H_2_O_2_ response of Tpx-roGFP2 and the kinetics of H_2_O_2_ detoxification in the Δ*ypdA* and Δ*brxAB* mutants. Interestingly, Tpx-roGFP2 showed a similar response to 100 mM H_2_O_2_ in all strains, but the Δ*ypdA* mutant was significantly impaired in H_2_O_2_ detoxification compared to the wild type (Figure 4E,F). These results clearly confirmed that the Δ*ypdA* mutant is defective to recover from oxidative stress due to its higher BSSB level resulting in an oxidized *E*_BSH_ as revealed using Brx-roGFP2 and thiol-metabolomics studies.

### *S*-Bacillithiolation of GapDH Is Not Affected in Δ*ypdA* and Δ*brxAB* Mutants or in *ypdA, brxA*, and *brxB* Complemented Strains

**FIGURE 5:**
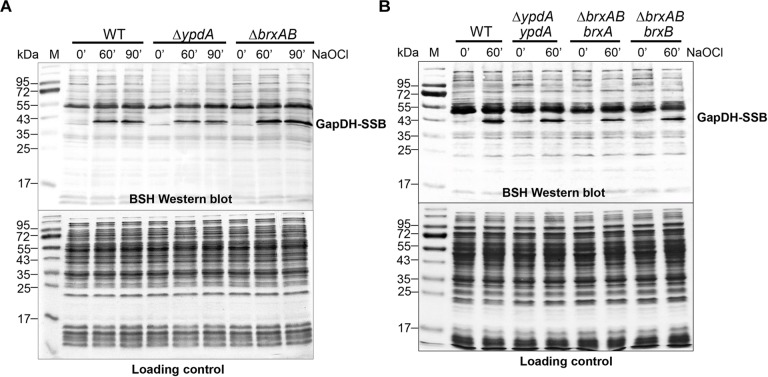
Protein *S*-bacillithiolation of GapDH is not affected in the Δ*ypdA* and Δ*brxAB* mutants **(A)** or in the *ypdA, brxA*, and *brxB* complemented strains **(B)** as revealed by non-reducing BSH Western blots. The prominent GapDH-SSB band is visible in the cell extracts of NaOCl-treated *S. aureus* cells using non-reducing BSH Western blots. Other bands visible under control and stress conditions are proteins cross-reactive with the polyclonal rabbit anti-BSH antibodies. The amount of GapDH-SSB is similar in the WT, Δ*ypdA* and Δ*brxAB* mutants **(A)** as well as in the *ypdA, brxA*, and *brxB* complemented strains **(B)**. The SDS PAGE loading control is shown at the bottom for comparison.

In *S. aureus*, the glyceraldehyde-3 phosphate dehydrogenase GapDH was previously identified as most abundant *S*-bacillithiolated protein under NaOCl stress that is visible as major band in BSH-specific non-reducing Western blots (Imber et al., 2018a). Since GapDH activity could be recovered with purified BrxA *in vitro* previously (Imber et al., 2018a), we analyzed the pattern of GapDH *S*-bacillithiolation in the Δ*brxAB* and Δ*ypdA* mutants as well as in *ypdA, brxA* and *brxB* complemented strains *in vivo*. However, the amount of *S*-bacillithiolated GapDH was similar after 100 μM NaOCl stress between wild type, Δ*brxAB* and Δ*ypdA* mutants and complemented strains (Figure 5A,B). This indicates that the absence of the BrxAB/YpdA pathway does not affect the level of *S*-bacillithiolation of GapDH under NaOCl stress.

### The Bacilliredoxins BrxA/B and the Putative BSSB Reductase YpdA Are Important for Growth and Survival Under Oxidative Stress and Macrophage Infections

**FIGURE 6:**
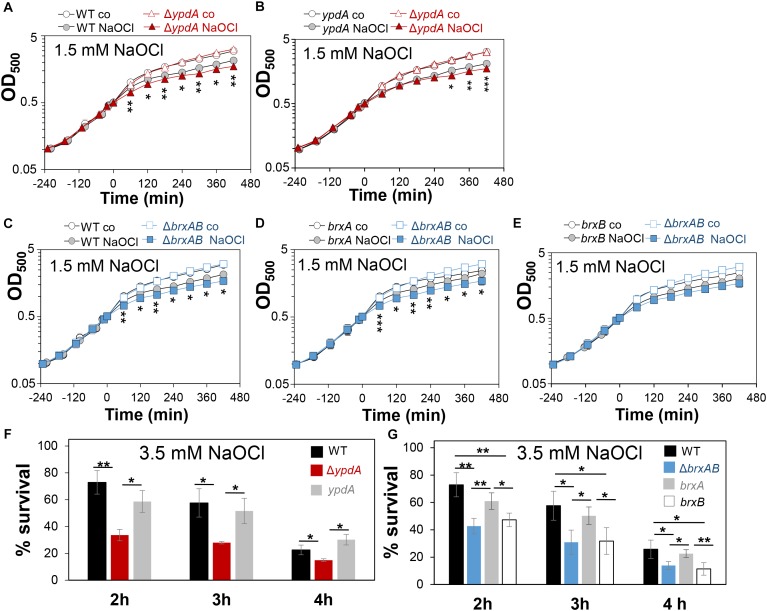
The *S. aureus*Δ*ypdA* and Δ*brxAB* mutants are more sensitive under NaOCl stress. **(A–E)** Growth curves of *S. aureus* COL WT, Δ*ypdA* and Δ*brxAB* mutants as well as *ypdA, brxA*, and *brxB* complemented strains in RPMI medium after exposure to 1.5 mM NaOCl stress at an OD_500_ of 0.5. **(F,G)** Survival rates were determined as CFUs for *S. aureus* COL WT, Δ*ypdA* and Δ*brxAB* mutants as well as *ypdA, brxA*, and *brxB* complemented strains at 2, 3, and 4 h after treatment with 3.5 mM NaOCl. Survival of the untreated control was set to 100%. Mean values and SD of 3–4 biological replicates are presented. The statistics was calculated using a Student’s unpaired two-tailed *t*-test by the graph prism software. Symbols are: ^ns^*p* > 0.05, ^∗^*p* ≤ 0.05, and ^∗∗^*p* ≤ 0.01.

**FIGURE 7:**
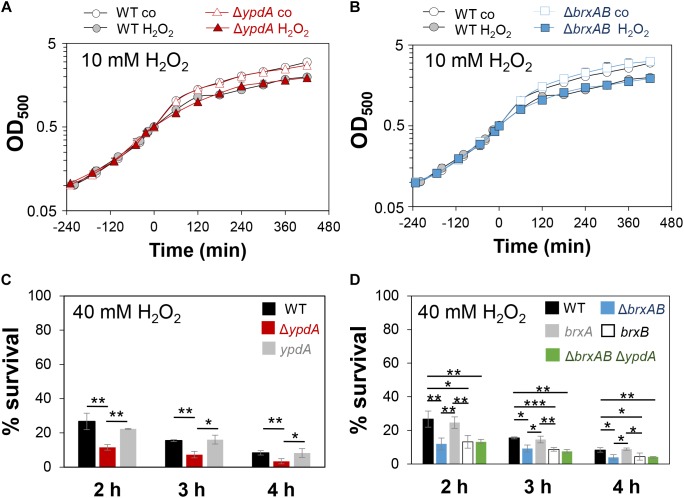
The *S. aureus*Δ*ypdA* and Δ*brxAB* mutants show increased sensitivities to H_2_O_2_ stress. **(A,B)** Growth curves of *S. aureus* COL WT, the Δ*ypdA* and Δ*brxAB* mutants in RPMI after exposure to 10 mM H_2_O_2_ stress at an OD_500_ of 0.5. **(C,D)** Survival rates were determined as CFUs for *S. aureus* COL WT, Δ*ypdA*, Δ*brxAB* and Δ*brxAB*Δ*ypdA* mutants as well as *ypdA, brxA, brxB* complemented strains at 2, 3, and 4 h after treatment with 40 mM H_2_O_2_. Survival of the untreated control was set to 100%. Mean values and SD of 3–5 biological replicates are presented. The statistics was calculated using a Student’s unpaired two-tailed *t*-test by the graph prism software. Symbols are: ^ns^*p* > 0.05, ^∗^*p* ≤ 0.05, and ^∗∗^*p* ≤ 0.01.

Next, we analyzed the physiological role of the BrxA/B/YpdA pathway for growth and survival of *S. aureus* under H_2_O_2_ and NaOCl stress. The growth of the Δ*ypdA* and Δ*brxAB* mutants in RPMI medium without stress exposure was comparable to the wild type (Figure 6A,C). Interestingly, both Δ*brxAB* and Δ*ypdA* mutants displayed a small, but statistically significant growth delay after exposure to sub-lethal amounts of 1.5 mM NaOCl compared to the wild type, while no growth delay was observed with sub-lethal 10 mM H_2_O_2_ (Figure 6A,C, 7A,B). This might indicate that BrxAB and YpdA function in the same pathway as already suggested by phylogenomic profiling using STRING search (Supplementary Figure S2). Determination of viable counts revealed significantly ∼2-fold decreased survival rates of both Δ*brxAB* and Δ*ypdA* mutants after exposure to lethal doses of 3.5 mM NaOCl and 40 mM H_2_O_2_ relative to the wild type (Figure 6F,G, 7C,D). These oxidant sensitive growth and survival phenotypes of the Δ*brxAB* and Δ*ypdA* mutants could be restored back to wild type levels by complementation with *brxA* and *ypdA*, respectively (Figure 6B,D,F,G, 7C,D). However, complementation of the Δ*brxAB* mutant with *brxB* did not restore the growth and viability of the wild type under NaOCl stress (Figure 6E,G), although xylose-inducible *brxB* expression of plasmid pRB473-*brxB* could be verified in Northern blots (Supplementary Figure S7). Moreover, the Δ*brxAB*Δ*ypdA* triple mutant displayed the same sensitivity as the Δ*brxAB* mutant to 40 mM H_2_O_2_ and 3 mM NaOCl indicating that BrxA and YpdA function in the same pathway for reduction of *S*-bacillithiolated proteins (Figure 7D and Supplementary Figure S8C).

**FIGURE 8:**
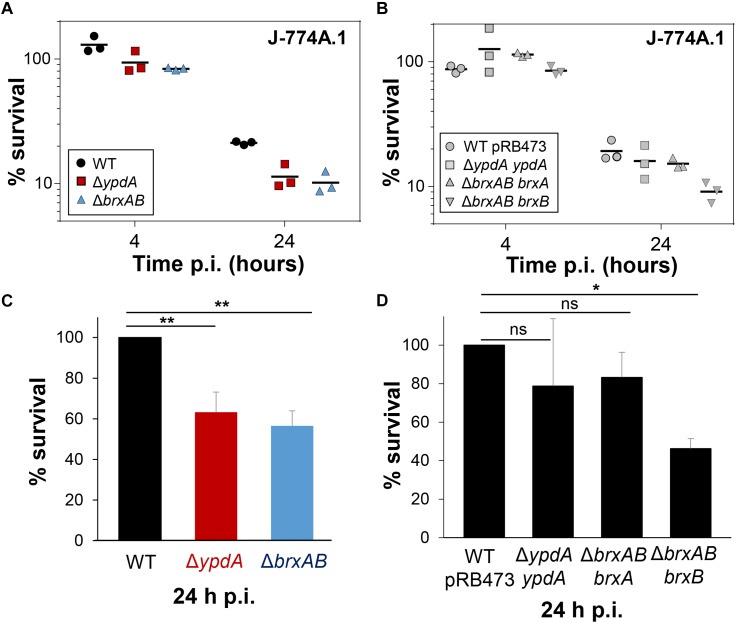
YpdA and BrxA/B promote the intracellular survival of *S. aureus* inside murine macrophages during infections. **(A,B)** The survival of *S. aureus* WT, Δ*ypdA* and Δ*brxAB* mutants and complemented strains was analyzed 2, 4 and 24 h post infection (p.i.) of the murine macrophage cell line J-774A.1 by CFU counting. The percentages in survival of the Δ*ypdA* and Δ*brxAB* mutants and complemented strains were calculated after 4 and 24 h in relation to the 2 h time point, which was set to 100%. **(C,D)** The average percentage in survival was calculated for Δ*ypdA* and Δ*brxAB* mutants **(C)** and complemented strain **(D)** in relation to the WT and WT with empty plasmid pRB473, which were set to 100%. Error bars represent the SEM and the statistics were calculated using one-way ANOVA and Tukey’s multiple comparisons *post hoc* test using the graph prism software (*p* = 0.0050 for WT/Δ*ypdA, p* = 0.0022 for WT/Δ*brxAB* and *p* = 0.026 for WT pRB473/Δ*brxAB brxB*). Symbols: ^ns^*p* > 0.05; ^∗^*p* ≤ 0.05, and ^∗∗^*p* ≤ 0.01.

To investigate the function of the BrxA/B/YpdA pathway under infection-relevant conditions, we measured the intracellular survival of the Δ*brxAB* and Δ*ypdA* mutants in phagocytosis assays inside murine macrophages of the cell line J-774A.1, as previously (Loi et al., 2018b). The viable counts (CFUs) of internalized *S. aureus* cells were determined at 2, 4, and 24 h post infection of the macrophages. The number of surviving cells decreased to 21.3% at 24 h post infection for the *S. aureus* COL wild type, but more strongly to 11.4 and 10.2% for the Δ*ypdA* and Δ*brxAB* mutants (Figure 8A,C). Thus, the number of viable counts was significantly ∼2-fold lower for both Δ*brxAB* and Δ*ypdA* mutants at 24 h post infection compared to the wild type. These sensitive phenotypes of the Δ*ypdA* and Δ*brxAB* mutants under macrophage infections could be restored to 80% of wild type levels after complementation with plasmid-encoded *ypdA* or *brxA*, respectively (Figure 8B,D). However, complementation with *brxB* did not restore the survival defect of the Δ*brxAB* mutant, pointing again to the major role of BrxA in this pathway.

Taken together, our results revealed that the bacilliredoxin BrxA and the putative BSSB reductase YpdA are required for improved survival of *S. aureus* inside macrophages to resist the oxidative burst. Our data suggest that BrxA and YpdA act together in the BrxA/BSH/YpdA pathway to regenerate *S*-bacillithiolated proteins and to restore the BSH redox potential upon recovery from oxidative stress during infections.

### The Flavin Disulfide Reductase YpdA Functions in BSSB Reduction and De-Bacillithiolation of GapDH-SSB in the BrxA/BSH/YpdA Electron Transfer Assay *in vitro*

**FIGURE 9:**
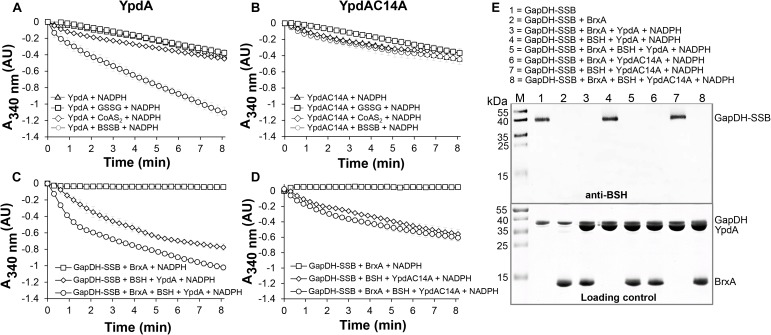
YpdA functions as BSSB reductase in the NADPH-coupled BrxA/BSH/YpdA electron pathway for de-bacillithiolation of GapDH-SSB *in vitro*. **(A)** Purified YpdA is able to reduce BSSB back to BSH with electrons from NADPH as measured by the absorbance change at 340 nm. Only little NADPH consumption was measured with YpdA alone in the absence of BSSB, with coenzymeA disulfide (CoAS2) or glutathione disulfide (GSSG) **(A)** and in the YpdAC14A mutant **(B)** indicating the function of the conserved Cys14 as active site of YpdA for BSSB reduction. **(C)** NADPH consumption of YpdA was measured in the coupled BrxA/BSH/YpdA de-bacillithiolation assay for reduction of GapDH-SSB. While fast NADPH consumption was measured upon de-bacillithiolation of GapDH-SSB with purified YpdA **(C)**, the reaction was much slower with the YpdAC14A mutant **(D)**. The coupled assays were conducted with 2.5 μM Gap-SSB, 12.5 μM BrxA, 40 μM BSH, 500 μM NADPH in 20 mM Tris, 1.25 mM EDTA, pH 8.0. After 30-min incubation, 12.5 μM YpdA or YpdAC14A proteins were added to the reaction mix and NADPH consumption was monitored at 340 nm as a function of time. Mean values and SEM of four independent experiments are shown. **(E)** The de-bacillithiolation of GapDH-SSB is catalyzed by BrxA or the complete BrxA/BSH/YpdA pathway, but not by YpdA alone as shown by non-reducing BSH-specific Western blots. The loading controls are shown below as Coomassie-stained SDS-PAGE with the same de-bacillithiolation reactions of GapDH-SSB as in the BSH-blot.

Next, we aimed to analyze the catalytic activity of purified YpdA in a NADPH-coupled assay with BSSB as substrate *in vitro*, since biochemical evidence for the function of YpdA as BSSB reductase activity *in vitro* is still missing (Mikheyeva et al., 2019). The His-tagged YpdA protein was purified as yellow colored enzyme and the UV-visible spectrum revealed the presence of the FAD co-factor indicated by the two absorbance peaks at 375 and 450 nm (Supplementary Figure S9). Incubation of YpdA protein with BSSB resulted in significant and fast consumption of NADPH as measured by a rapid absorbance decrease at 340 nm (Figure 9A). Only little NADPH consumption was measured with YpdA alone in the absence of the BSSB substrate supporting previous finding that YpdA consumes NADPH alone (Mikheyeva et al., 2019). However, in our assays, BSSB significantly enhanced NADPH consumption by YpdA compared to the control reaction without BSSB. No increased NADPH consumption was measured with coenzyme A disulphide (CoAS_2_) or GSSG as substrate indicating the specificity of YpdA for BSSB (Figure 9A). In addition, we investigated the role of the conserved Cys14 of YpdA for the BSSB reductase activity in the NADPH-coupled assay. NADPH-consumption of YpdAC14A upon BSSB reduction was much slower and similar to the control reaction of YpdA and YpdAC14A without BSSB (Figure 9B).

Our *in vivo* data support that YpdA and BrxA act together in the BrxA/BSH/YpdA de-bacillithiolation pathway. Thus, we analyzed NADPH-consumption by the BrxA/BSH/YpdA electron pathway in de-bacillithiolation of GapDH-SSB *in vitro*. The de-bacillithiolation assays revealed fast NADPH consumption in the complete BrxA/BSH/YpdA coupled assays (Figure 9C). NADPH consumption by YpdA was slower in the absence of BrxA and might be caused by residual BSSB in the BSH samples. The control reaction of GapDH-SSB with BrxA did not consume NADPH and only little NADPH consumption was measured with BrxA, BSH and the YpdAC14A mutant protein in de-bacillithiolation of GapDH-SSB (Figure 9D).

In addition, BSH-specific non-reducing Western blots were used to investigate if BrxA and the complete BrxA/BSH/YpdA pathway catalyze de-bacillithiolation of GapDH-SSB (Figure 9E). The BSH-blots showed that BrxA is sufficient for de-bacillithiolation of GapDH-SSB, since all reactions of GapDH-SSB with BrxA lead to complete de-bacillithiolation with and without YpdA or YpdAC14A plus NADPH. However, the reactions of GapDH-SSB with YpdA/NADPH alone did not lead to reduction of GapDH-SSB, indicating the main role of BrxA in de-bacillithiolation while YpdA functions in regeneration of BSH in the BrxA/BSH/YpdA/NADPH redox cycle.

In conclusion, our biochemical assays revealed that YpdA functions as BSSB reductase in an NADPH coupled assay. Cys14 of YpdA is important for the BSSB reductase activity *in vitro*. Thus, YpdA facilitates together with BrxA the reduction of *S*-bacillithiolated GapDH in the BrxA/BSH/YpdA redox pathway upon recovery from oxidative stress.

## Discussion

The putative disulfide reductase YpdA was previously shown to be phylogenetically associated with the BSH biosynthesis enzymes and bacilliredoxins (Supplementary Figure S2), providing evidence for a functional Brx/BSH/YpdA pathway in BSH-producing bacteria (Gaballa et al., 2010). Recent work confirmed the importance of YpdA for the BSH/BSSB redox balance and survival under oxidative stress and neutrophil infections in *S. aureus in vivo* (Mikheyeva et al., 2019). Here, we have studied the role of the bacilliredoxins BrxA/B and the BSSB reductase YpdA in the defense of *S. aureus* against oxidative stress *in vivo* and their biochemical function in the de-bacillithiolation pathway *in vitro*. Transcription of *brxA, brxB* and *ypdA* is strongly upregulated under disulfide stress, provoked by diamide and NaOCl. About two to fourfold increased transcription of *ypdA, brxA*, and *brxB* was previously found under H_2_O_2_, diamide and NaOCl stress, by the antimicrobial surface coating composed of Ag^+^ and Ru^+^ (AGXX^®^) and after exposure to azurophilic granule proteins in *S. aureus* (Palazzolo-Ballance et al., 2008; Posada et al., 2014; Mäder et al., 2016; Loi et al., 2018a,b; Mikheyeva et al., 2019). The elevated transcription of *brxA, brxB*, and *ypdA* under disulfide stress correlated with the up-regulation of the *bshA, bshB*, and *bshC* genes for BSH biosynthesis in *S. aureus* and *B. subtilis* (Chi et al., 2011; Nicolas et al., 2012; Loi et al., 2018a,b). The *bshA, bshB*, and *bshC* genes and operons are under control of the disulfide stress-specific Spx regulator in *B. subtilis*, which controls a large regulon for thiol-redox homeostasis (Gaballa et al., 2013). Thus, genes for BSH biosynthesis and the BrxA/B/YpdA pathway might be also regulated by Spx in *S. aureus*.

The co-regulation of BrxA/B and YpdA under disulfide stress points to their function in the same pathway in *S. aureus*. HOCl, diamide and AGXX^®^ were shown to cause a strong disulfide stress response in the transcriptome and protein *S*-bacillithiolation in the proteome of *S. aureus* (Imber et al., 2018a; Loi et al., 2018a,b). Thus, the BrxA/B and YpdA redox enzymes are up-regulated under conditions of protein *S*-bacillithiolations, connecting their functions to the de-bacillithiolation pathway. We could show here that NaOCl stress leads to five to sixfold depletion of the cellular pool of reduced BSH in the *S. aureus* COL wild type, which was not accompanied by an enhanced BSSB level. In the previous study, 20 mM H_2_O_2_ resulted in twofold reduction of BSH and threefold increase of BSSB in the *S. aureus* wild type (Mikheyeva et al., 2019). Most probably, the increased BSSB level under NaOCl stress was used for protein *S*-bacillithiolation in our study (Imber et al., 2018a), while sub-lethal 20 mM H_2_O_2_ might not lead to an increase in *S-*bacillithiolation in the previous study (Mikheyeva et al., 2019).

The BSH/BSSB redox ratio of *S. aureus* wild type cells was determined as ∼35:1 under control conditions and decreased threefold to 10:1 under NaOCl. Of note, this basal BSH/BSSB ratio in *S. aureus* COL wild type was higher compared to the basal BSH/BSSB ratio of ∼17:1 as determined previously in the *bshC* repaired SH1000 strain (Mikheyeva et al., 2019). In *E. coli*, the GSH/GSSG redox ratio was determined in the range between 30:1 and 100:1 (Hwang et al., 1995; Van Laer et al., 2013), which is similar as measured for the basal BSH/BSSB ratio in *S. aureus* COL. The differences in the BSH/BSSB ratios might be related to different *S. aureus* strain backgrounds or growth conditions. Nevertheless, NaOCl and H_2_O_2_ decreased the BSH/BSSB ratio in our and the previous study (Mikheyeva et al., 2019). In the *S. aureus* Δ*brxAB* mutant, we also measured a threefold decrease of the BSH/BSSB ratio from control conditions (38:1) to NaOCl (12:1). However, the Δ*ypdA* mutant showed a twofold enhanced BSSB level in control and NaOCl-treated cells, leading to a significantly decreased BSH/BSSB ratio under control (17:1) and NaOCl stress (5:1). These results support previous results of the *bshC* repaired SH1000, showing a decreased BSH/BSSB ratio under control (6:1) to H_2_O_2_ stress (2:1) (Mikheyeva et al., 2019), although both ratios were again much lower as in our study. Taken together, our data indicate that BrxAB are dispensable for the BSH redox homeostasis, while YpdA is essential for BSSB reduction to maintain the reduced pool of BSH and a high BSH/BSSB ratio in *S. aureus*.

Brx-roGFP2 biosensor measurements provide further support that YpdA is the candidate BSSB reductase. The Δ*ypdA* mutant was significantly impaired to restore reduced *E*_BSH_ during recovery from NaOCl and H_2_O_2_ stress as calculated using the Nernst equation based on the OxD values of the Brx-roGFP2 biosensor measurements (Supplementary Tables S5, S6). Moreover, application of the Tpx-roGFP2 biosensor revealed a delay in H_2_O_2_ detoxification in Δ*ypdA* mutant cells during the recovery phase. These results clearly support the important role of YpdA as BSSB reductase particularly under oxidative stress to recover reduced *E*_BSH_ required for detoxification of ROS.

These *in vivo* data were further corroborated by biochemical activity assays of YpdA for BSSB reduction in a NADPH-coupled assay. While little NADPH consumption was measured in the presence of YpdA alone, BSSB significantly enhanced NADPH consumption, supporting the crucial role of YpdA as BSSB reductase *in vitro*. Further electron transfer assays revealed that YpdA functions together with BrxA and BSH in reduction of GapDH-SSB *in vitro*. Previous de-bacillithiolation assays have revealed regeneration of GapDH activity by BrxA *in vitro* (Imber et al., 2018a). Here, we confirmed that BrxA activity is sufficient for complete de-bacillithiolation of GapDH-SSB *in vitro*, while YpdA alone had no effect on the GapDH-SSB reduction. Thus, BrxA catalyzes reduction of *S*-bacillithiolated proteins and YpdA is involved in BSH regeneration in the complete BrxA/BSH/YpdA redox cycle.

The BSSB reductase activity of YpdA was shown to be dependent on the conserved Cys14, which is located in the glycine-rich Rossmann-fold NAD(P)H binding domain (GGGPC_14_G) (Bragg et al., 1997; Mikheyeva et al., 2019). Cys14 might be *S*-bacillithiolated by BSSB and reduced by electron transfer from NADPH via the FAD co-factor. Cys14 was previously identified as oxidized under NaOCl stress in the *S. aureus* redox proteome using the OxICAT method, further supporting its role as active site Cys and its *S*-bacillithiolation during the BrxA/BSH/YpdA catalytic cycle (Imber et al., 2018a). The catalytic mechanism of BSSB reduction via Cys14 of YpdA is an interesting subject of future studies.

Previous phenotype results of the Δ*ypdA* mutant revealed that YpdA is important for survival of *S. aureus* in infection assays with human neutrophils (Mikheyeva et al., 2019). Our phenotype analyses further showed protective functions of the complete BrxA/BSH/YpdA redox pathway for growth and survival of *S. aureus* under oxidative stress *in vitro* and in macrophage infections *in vivo*. The Δ*ypdA* and Δ*brxAB* mutants were significantly impaired in growth and survival after exposure to sub-lethal and lethal doses of NaOCl and displayed survival defects under lethal H_2_O_2_. Moreover, the H_2_O_2_ and NaOCl-sensitivity and the defect to recover reduced *E*_BSH_ in the Δ*brxAB*Δ*ypdA* triple mutant was comparable with that of the Δ*ypdA* mutant (Figure 7D and Supplementary Figure S8). These results clearly indicate that BrxA/B and YpdA function in the same de-bacillithiolation pathway, which is an important defense mechanism of *S. aureus* against oxidative stress.

Based on previous bacilliredoxin activity assays *in vitro*, both BrxA and BrxB should use a monothiol mechanism to reduce *S*-bacillithiolated client proteins, such as OhrR, GapDH and MetE in *B. subtilis* and *S. aureus* (Gaballa et al., 2014; Imber et al., 2018a). Most di-thiol Grx of *E. coli* (Grx1, Grx2, and Grx3) use the monothiol mechanism for de-glutathionylation of proteins (Lillig et al., 2008; Allen and Mieyal, 2012; Loi et al., 2015). In the monothiol mechanism, the nucleophilic thiolate of the Brx CGC motif attacks the *S*-bacillithiolated protein, resulting in reduction of the protein substrate and Brx-SSB formation. Brx-SSB is then recycled by BSH, leading to increased BSSB formation. YpdA reduces BSSB back to BSH with electrons from NADPH (Figure 1B). The oxidation-sensitive phenotypes of Δ*ypdA* and Δ*brxAB* mutants could be complemented by plasmid-encoded *ypdA* and *brxA*, but not *brxB*, respectively. These results provide evidence for the function of the BrxA/BSH/YpdA de-bacillithiolation pathway using the monothiol-Brx mechanism in *S. aureus*.

Similar phenotypes were found for mutants lacking related redox enzymes of the GSH and mycothiol pathways in other bacteria. In *E. coli*, strains lacking the Gor and Grx are more sensitive under diamide and cumene hydroperoxide stress (Alonso-Moraga et al., 1987; Vlamis-Gardikas et al., 2002; Lillig et al., 2008). In *Mycobacterium smegmatis*, the mycoredoxin-1 mutant displayed an oxidative stress-sensitive phenotype (Van Laer et al., 2012). In *Corynebacterium glutamicum*, deficiency of the Mtr resulted in an oxidized mycothiol redox potential (Tung et al., 2019), and Mtr overexpression contributed to improved oxidative stress resistance (Si et al., 2016). Taken together, our results revealed that not only BSH, but also BrxA and YpdA are required for virulence and promote survival in infection assays inside murine macrophages.

In several human pathogens, such as *Streptococcus pneumoniae, Listeria monocytogenes, Salmonella Typhimurium*, and *Pseudomonas aeruginosa*, LMW thiols or the Gor are required for virulence, colonization and to resist host-derived oxidative or nitrosative stress (Potter et al., 2012; Song et al., 2013; Reniere et al., 2015; Tung et al., 2018; Wongsaroj et al., 2018). *S. aureus* BSH deficient mutants showed decreased survival in murine macrophages and in human whole blood infections (Pöther et al., 2013; Posada et al., 2014). The virulence mechanisms might be related to a lack of BSH regeneration and decreased recovery of inactivated *S*-bacillithiolated proteins inside macrophages. Future studies should elucidate the targets for *S*-bacillithiolations that are reduced by the BrxA/BSH/YpdA pathway inside macrophages, increasing survival, metabolism or persistence under infections.

In summary, our results showed the importance of the BrxA/BSH/YpdA redox pathway to resist oxidative stress and macrophage infection in *S. aureus*. Through measurements of the BSH/BSSB redox ratio and *E*_BSH_, we provide evidence that the NADPH-dependent disulfide reductase YpdA regenerates BSH and restores reduced *E*_BSH_ upon recovery from oxidative stress in *S. aureus*. Finally, biochemical evidence for YpdA as BSSB reductase and for the role of BrxA/BSH/YpdA pathway in de-bacillithiolation was provided *in vitro*. The detailed biochemical mechanism of YpdA and the cross-talk of the Trx and Brx systems in de-bacillithiolation under oxidative stress and infections are subject of our future studies.

## Author Contributions

HA and NL designed the experiments of this study. NL, VVL, VNF, QNT and SS constructed the mutants, performed the experiments and analyzed the data of this manuscript. MW and RH performed the HPLC thiol metabolomics analyses and analyzed the data. KT and MF contributed with the infection assays to this work. CH synthesized BSH and BSSB for the biochemical assays of the manuscript. NL and HA wrote the manuscript. All authors contributed with corrections of the manuscript.

## Conflict of Interest Statement

The authors declare that the research was conducted in the absence of any commercial or financial relationships that could be construed as a potential conflict of interest.

## Abbreviations

BSH: bacillithiol
BSSB: bacillithiol disulfide
BrxA/B: bacilliredoxin A (YphP)/bacilliredoxin B (YqiW)
CFUs: colony forming units
DTT: dithiothreitol
*E*_BSH_: bacillithiol redox potential
GapDH: glyceraldehyde 3-phosphate dehydrogenase
GSH: glutathione
GSSG: glutathione disulfide
Gor: glutathione disulfide reductase
Grx: glutaredoxins
HOCl: hypochlorous acid
LMW: low molecular weight
Mtr: mycothiol disulfide reductase
NaOCl: sodium hypochlorite
OD_500_: optical density at 500 nm
rdw: raw dry weight
RCS: reactive chlorine species
ROS: reactive oxygen species
YpdA: bacillithiol disulfide reductase

## References

[B1] Allen E. M. Mieyal J. J. (2012). Protein-thiol oxidation and cell death: regulatory role of glutaredoxins. *Antioxid. Redox Signal.* 17 1748–1763. 10.1089/ars.2012.4644 22530666 PMC3474186

[B2] Alonso-Moraga A. Bocanegra A. Torres J. M. Lopez-Barea J. Pueyo C. (1987). Glutathione status and sensitivity to GSH-reacting compounds of *Escherichia coli* strains deficient in glutathione metabolism and/or catalase activity. *Mol. Cell Biochem.* 73 61–68. 3543652 10.1007/BF00229377

[B3] Archer G. L. (1998). *Staphylococcus aureus*: a well-armed pathogen. *Clin. Infect. Dis.* 26 1179–1181. 10.1086/520289 9597249

[B4] Argyrou A. Blanchard J. S. (2004). Flavoprotein disulfide reductases: advances in chemistry and function. *Progr. Nucleic Acid Res. Mol. Biol.* 78 89–142. 10.1016/s0079-6603(04)78003-4 15210329

[B5] Arnaud M. Chastanet A. Débarbouillé M. (2004). New vector for efficient allelic replacement in naturally nontransformable, low-GC-content, gram-positive bacteria. *Appl. Environ. Microbiol.* 70 6887–6891. 10.1128/aem.70.11.6887-6891.2004 15528558 PMC525206

[B6] Beavers W. N. Skaar E. P. (2016). Neutrophil-generated oxidative stress and protein damage in *Staphylococcus aureus*. *Pathog. Dis.* 74:ftw060. 10.1093/femspd/ftw060 27354296 PMC5975594

[B7] Boucher H. W. Corey G. R. (2008). Epidemiology of methicillin-resistant *Staphylococcus aureus*. *Clin. Infect Dis.* 46(Suppl. 5), S344–S349. 10.1086/533590 18462089

[B8] Bragg P. D. Glavas N. A. Hou C. (1997). Mutation of conserved residues in the NADP(H)-binding domain of the proton translocating pyridine nucleotide transhydrogenase of *Escherichia coli*. *Arch. Biochem. Biophys.* 338 57–66. 10.1006/abbi.1996.9797 9015388

[B9] Brückner R. Wagner E. Götz F. (1993). Characterization of a sucrase gene from *Staphylococcus xylosus*. *J. Bacteriol.* 175 851–857. 10.1128/jb.175.3.851-857.1993 8423155 PMC196229

[B10] Chandrangsu P. Loi V. V. Antelmann H. Helmann J. D. (2018). The role of bacillithiol in Gram-positive *Firmicutes*. *Antioxid. Redox Signal.* 28 445–462. 10.1089/ars.2017.7057 28301954 PMC5790435

[B11] Chi B. K. Gronau K. Mäder U. Hessling B. Becher D. Antelmann H. (2011). *S*-bacillithiolation protects against hypochlorite stress in *Bacillus subtilis* as revealed by transcriptomics and redox proteomics. *Mol. Cell Proteo.* 10:M111009506. 10.1074/mcp.M111.009506 21749987 PMC3226405

[B12] Chi B. K. Roberts A. A. Huyen T. T. Bäsell K. Becher D. Albrecht D. (2013). *S*-bacillithiolation protects conserved and essential proteins against hypochlorite stress in *Firmicutes* bacteria. *Antioxid. Redox Signal.* 18 1273–1295. 10.1089/ars.2012.4686 22938038 PMC3584511

[B13] Deponte M. (2013). Glutathione catalysis and the reaction mechanisms of glutathione-dependent enzymes. *Biochim. Biophys. Acta* 1830 3217–3266. 10.1016/j.bbagen.2012.09.018 23036594

[B14] Dooley C. T. Dore T. M. Hanson G. T. Jackson W. C. Remington S. J. Tsien R. Y. (2004). Imaging dynamic redox changes in mammalian cells with green fluorescent protein indicators. *J. Biol. Chem.* 279 22284–22293. 10.1074/jbc.m312847200 14985369

[B15] Fahey R. C. (2013). Glutathione analogs in prokaryotes. *Biochim. Biophys. Acta* 1830 3182–3198. 10.1016/j.bbagen.2012.10.006 23075826

[B16] Fuangthong M. Atichartpongkul S. Mongkolsuk S. Helmann J. D. (2001). OhrR is a repressor of *ohrA*, a key organic hydroperoxide resistance determinant in *Bacillus subtilis*. *J. Bacteriol.* 183 4134–4141. 10.1128/jb.183.14.4134-4141.2001 11418552 PMC95301

[B17] Gaballa A. Antelmann H. Hamilton C. J. Helmann J. D. (2013). Regulation of *Bacillus subtilis* bacillithiol biosynthesis operons by Spx. *Microbiology* 159 2025–2035. 10.1099/mic.0.070482-0 23894131

[B18] Gaballa A. Chi B. K. Roberts A. A. Becher D. Hamilton C. J. Antelmann H. (2014). Redox regulation in *Bacillus subtilis*: The bacilliredoxins BrxA(YphP) and BrxB(YqiW) function in de-bacillithiolation of S-bacillithiolated OhrR and MetE. *Antioxid. Redox Signal.* 21 357–367. 10.1089/ars.2013.5327 24313874 PMC4076974

[B19] Gaballa A. Newton G. L. Antelmann H. Parsonage D. Upton H. Rawat M. (2010). Biosynthesis and functions of bacillithiol, a major low-molecular-weight thiol in Bacilli. *Proc. Natl. Acad. Sci. U.S.A.* 107 6482–6486. 10.1073/pnas.1000928107 20308541 PMC2851989

[B20] Hillion M. Antelmann H. (2015). Thiol-based redox switches in prokaryotes. *Biol. Chem.* 396 415–444. 10.1515/hsz-2015-0102 25720121 PMC4438307

[B21] Hiras J. Sharma S. V. Raman V. Tinson R. A. J. Arbach M. Rodrigues D. F. (2018). Physiological studies of *Chlorobiaceae* suggest that bacillithiol derivatives are the most widespread thiols in bacteria. *MBio* 9:e01603-18. 10.1128/mBio.01603-18 30482829 PMC6282198

[B22] Hwang C. Lodish H. F. Sinskey A. J. (1995). Measurement of glutathione redox state in cytosol and secretory pathway of cultured cells. *Methods Enzymol.* 251 212–221. 10.1016/0076-6879(95)51123-7 7651199

[B23] Imber M. Huyen N. T. T. Pietrzyk-Brzezinska A. J. Loi V. V. Hillion M. Bernhardt J. (2018a). Protein *S*-bacillithiolation functions in thiol protection and redox regulation of the glyceraldehyde-3-phosphate dehydrogenase Gap in *Staphylococcus aureus* under hypochlorite stress. *Antioxid. Redox Signal.* 28 410–430. 10.1089/ars.2016.6897 27967218 PMC5791933

[B24] Imber M. Loi V. V. Reznikov S. Fritsch V. N. Pietrzyk-Brzezinska A. J. Prehn J. (2018b). The aldehyde dehydrogenase AldA contributes to the hypochlorite defense and is redox-controlled by protein *S*-bacillithiolation in *Staphylococcus aureus*. *Redox. Biol.* 15 557–568. 10.1016/j.redox.2018.02.001 29433022 PMC5975064

[B25] Imber M. Pietrzyk-Brzezinska A. J. Antelmann H. (2018c). Redox regulation by reversible protein *S*-thiolation in Gram-positive bacteria. *Redox. Biol.* 20 130–145. 10.1016/j.redox.2018.08.017 30308476 PMC6178380

[B26] Lee J. W. Soonsanga S. Helmann J. D. (2007). A complex thiolate switch regulates the *Bacillus subtilis* organic peroxide sensor OhrR. *Proc. Natl. Acad. Sci. U.S.A.* 104 8743–8748. 10.1073/pnas.0702081104 17502599 PMC1885573

[B27] Lillig C. H. Berndt C. Holmgren A. (2008). Glutaredoxin systems. *Biochim. Biophys. Acta* 1780 1304–1317. 10.1016/j.bbagen.2008.06.003 18621099

[B28] Livermore D. M. (2000). Antibiotic resistance in staphylococci. *Int. J. Antimicrob. Agents* 16(Suppl. 1), S3–S10. 11137402 10.1016/s0924-8579(00)00299-5

[B29] Loi V. V. Busche T. Preuss T. Kalinowski J. Bernhardt J. Antelmann H. (2018a). The AGXX antimicrobial coating causes a thiol-specific oxidative stress response and protein S-bacillithiolation in *Staphylococcus aureus*. *Front. Microbiol.* 9:3037. 10.3389/fmicb.2018.03037 30619128 PMC6299908

[B30] Loi V. V. Busche T. Tedin K. Bernhardt J. Wollenhaupt J. Huyen N. T. T. (2018b). Redox-sensing under hypochlorite stress and infection conditions by the Rrf2-family repressor HypR in *Staphylococcus aureus*. *Antioxid. Redox Signal.* 29 615–636. 10.1089/ars.2017.7354 29237286 PMC6067689

[B31] Loi V. V. Harms M. Müller M. Huyen N. T. T. Hamilton C. J. Hochgräfe F. (2017). Real-time imaging of the bacillithiol redox potential in the human pathogen *Staphylococcus aureus* using a genetically encoded bacilliredoxin-fused redox biosensor. *Antioxid. Redox Signal.* 26 835–848. 10.1089/ars.2016.6733 27462976 PMC5444506

[B32] Loi V. V. Rossius M. Antelmann H. (2015). Redox regulation by reversible protein *S*-thiolation in bacteria. *Front. Microbiol.* 6:187 10.3389/fmicb.2015.00187 PMC4360819 25852656

[B33] Lowy F. D. (1998). *Staphylococcus aureus* infections. *N. Engl. J. Med.* 339 520–532. 9709046 10.1056/NEJM199808203390806

[B34] Mäder U. Nicolas P. Depke M. Pane-Farre J. Debarbouille M. Van Der Kooi-Pol M. M. (2016). *Staphylococcus aureus* transcriptome architecture: from laboratory to infection-mimicking conditions. *PLoS Genet.* 12:e1005962. 10.1371/journal.pgen.1005962 27035918 PMC4818034

[B35] Mikheyeva I. V. Thomas J. M. Kolar S. L. Corvaglia A. R. Gaiotaa N. Leo S. (2019). YpdA, a putative bacillithiol disulfide reductase, contributes to cellular redox homeostasis and virulence in *Staphylococcus aureus*. *Mol. Microbiol.* 111 1039–1056. 10.1111/mmi.14207 30636083 PMC6458089

[B36] Newton G. L. Fahey R. C. Rawat M. (2012). Detoxification of toxins by bacillithiol in *Staphylococcus aureus*. *Microbiology* 158 1117–1126. 10.1099/mic.0.055715-0 22262099 PMC3949421

[B37] Nicolas P. Mäder U. Dervyn E. Rochat T. Leduc A. Pigeonneau N. (2012). Condition-dependent transcriptome reveals high-level regulatory architecture in *Bacillus subtilis*. *Science* 335 1103–1106. 10.1126/science.1206848 22383849

[B38] Palazzolo-Ballance A. M. Reniere M. L. Braughton K. R. Sturdevant D. E. Otto M. Kreiswirth B. N. (2008). Neutrophil microbicides induce a pathogen survival response in community-associated methicillin-resistant *Staphylococcus aureus*. *J. Immunol.* 180 500–509. 10.4049/jimmunol.180.1.500 18097052

[B39] Pendleton J. N. Gorman S. P. Gilmore B. F. (2013). Clinical relevance of the ESKAPE pathogens. *Expert Rev. Anti. Infect. Ther.* 11 297–308. 10.1586/eri.13.12 23458769

[B40] Posada A. C. Kolar S. L. Dusi R. G. Francois P. Roberts A. A. Hamilton C. J. (2014). Importance of bacillithiol in the oxidative stress response of *Staphylococcus aureus*. *Infect. Immun.* 82 316–332. 10.1128/IAI.01074-13 24166956 PMC3911838

[B41] Pöther D. C. Gierok P. Harms M. Mostertz J. Hochgräfe F. Antelmann H. (2013). Distribution and infection-related functions of bacillithiol in *Staphylococcus aureus*. *Int. J. Med. Microbiol.* 303 114–123. 10.1016/j.ijmm.2013.01.003 23517692

[B42] Potter A. J. Trappetti C. Paton J. C. (2012). *Streptococcus pneumoniae* uses glutathione to defend against oxidative stress and metal ion toxicity. *J. Bacteriol.* 194 6248–6254. 10.1128/JB.01393-12 22984260 PMC3486410

[B43] Reniere M. L. Whiteley A. T. Hamilton K. L. John S. M. Lauer P. Brennan R. G. (2015). Glutathione activates virulence gene expression of an intracellular pathogen. *Nature* 517 170–173. 10.1038/nature14029 25567281 PMC4305340

[B44] Rosenblum E. D. Tyrone S. (1964). Serology, density, and morphology of staphylococcal phages. *J. Bacteriol.* 88 1737–1742. 14240964 10.1128/jb.88.6.1737-1742.1964 PMC277480

[B45] Si M. Zhao C. Zhang B. Wei D. Chen K. Yang X. (2016). Overexpression of mycothiol disulfide reductase enhances *Corynebacterium glutamicum* robustness by modulating cellular redox homeostasis and antioxidant proteins under oxidative stress. *Sci. Rep.* 6:29491. 10.1038/srep29491 27383057 PMC4935862

[B46] Song M. Husain M. Jones-Carson J. Liu L. Henard C. A. Vazquez-Torres A. (2013). Low-molecular-weight thiol-dependent antioxidant and antinitrosative defences in *Salmonella* pathogenesis. *Mol. Microbiol.* 87 609–622. 10.1111/mmi.12119 23217033 PMC3885168

[B47] Tam le T. Eymann C. Albrecht D. Sietmann R. Schauer F. Hecker M. (2006). Differential gene expression in response to phenol and catechol reveals different metabolic activities for the degradation of aromatic compounds in *Bacillus subtilis*. *Environ. Microbiol.* 8 1408–1427. 10.1111/j.1462-2920.2006.01034.x 16872404

[B48] Tung Q. N. Linzner N. Loi V. V. Antelmann H. (2018). Application of genetically encoded redox biosensors to measure dynamic changes in the glutathione, bacillithiol and mycothiol redox potentials in pathogenic bacteria. *Free Radic. Biol. Med.* 128 84–96. 10.1016/j.freeradbiomed.2018.02.018 29454879

[B49] Tung Q. N. Loi V. V. Busche T. Nerlich A. Mieth M. Milse J. (2019). Stable integration of the Mrx1-roGFP2 biosensor to monitor dynamic changes of the mycothiol redox potential in *Corynebacterium glutamicum*. *Redox. Biol.* 20 514–525. 10.1016/j.redox.2018.11.012 30481728 PMC6258114

[B50] Van Laer K. Buts L. Foloppe N. Vertommen D. Van Belle K. Wahni K. (2012). Mycoredoxin-1 is one of the missing links in the oxidative stress defence mechanism of Mycobacteria. *Mol. Microbiol.* 86 787–804. 10.1111/mmi.12030 22970802

[B51] Van Laer K. Hamilton C. J. Messens J. (2013). Low-molecular-weight thiols in thiol-disulfide exchange. *Antioxid. Redox Signal.* 18 1642–1653. 10.1089/ars.2012.4964 23075082

[B52] Vlamis-Gardikas A. Potamitou A. Zarivach R. Hochman A. Holmgren A. (2002). Characterization of *Escherichia coli* null mutants for glutaredoxin 2. *J. Biol. Chem.* 277 10861–10868. 11741965 10.1074/jbc.M111024200

[B53] Wetzstein M. Völker U. Dedio J. Löbau S. Zuber U. Schiesswohl M. (1992). Cloning, sequencing, and molecular analysis of the *dnaK* locus from *Bacillus subtilis*. *J. Bacteriol.* 174 3300–3310. 10.1128/jb.174.10.3300-3310.1992 1339421 PMC205999

[B54] Winterbourn C. C. Kettle A. J. (2013). Redox reactions and microbial killing in the neutrophil phagosome. *Antioxid. Redox Signal.* 18 642–660. 10.1089/ars.2012.4827 22881869

[B55] Winterbourn C. C. Kettle A. J. Hampton M. B. (2016). Reactive oxygen species and neutrophil function. *Annu. Rev. Biochem.* 85 765–792. 10.1146/annurev-biochem-060815-014442 27050287

[B56] Wongsaroj L. Saninjuk K. Romsang A. Duang-Nkern J. Trinachartvanit W. Vattanaviboon P. (2018). *Pseudomonas aeruginosa* glutathione biosynthesis genes play multiple roles in stress protection, bacterial virulence and biofilm formation. *PLoS One* 13:e0205815. 10.1371/journal.pone.0205815 30325949 PMC6191110

