# Supplementary material for: Staphylococcus aureus Uses the Bacilliredoxin (BrxAB)/Bacillithiol Disulfide Reductase (YpdA) Redox Pathway to Defend Against Oxidative Stress Under Infections
Source: Front Microbiol. 2019 Jun 18;10:1355. doi: 10.3389/fmicb.2019.01355 (PMC6591457; doi:10.3389/fmicb.2019.01355)
Supplement: Supplementary file 1 [file Data_Sheet_1.PDF]

# Figure S1

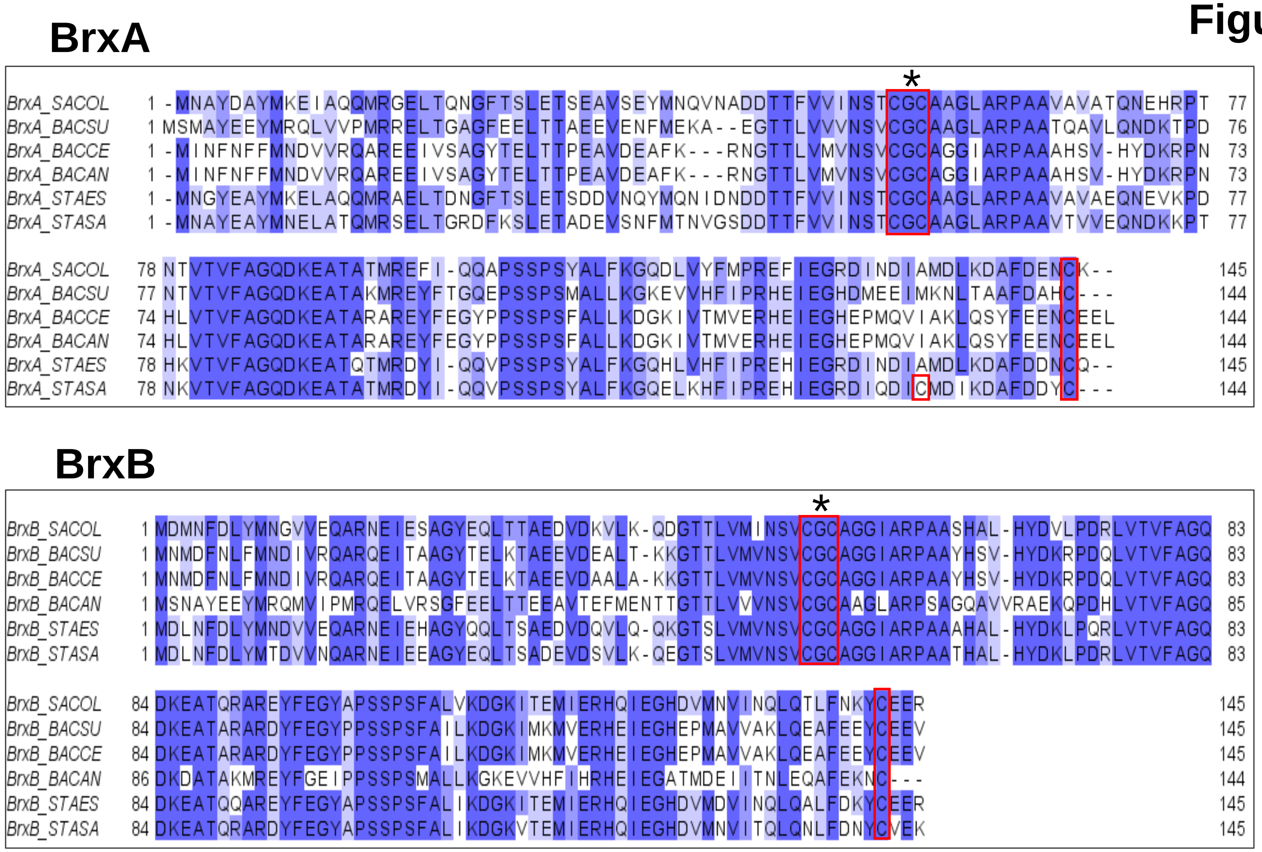


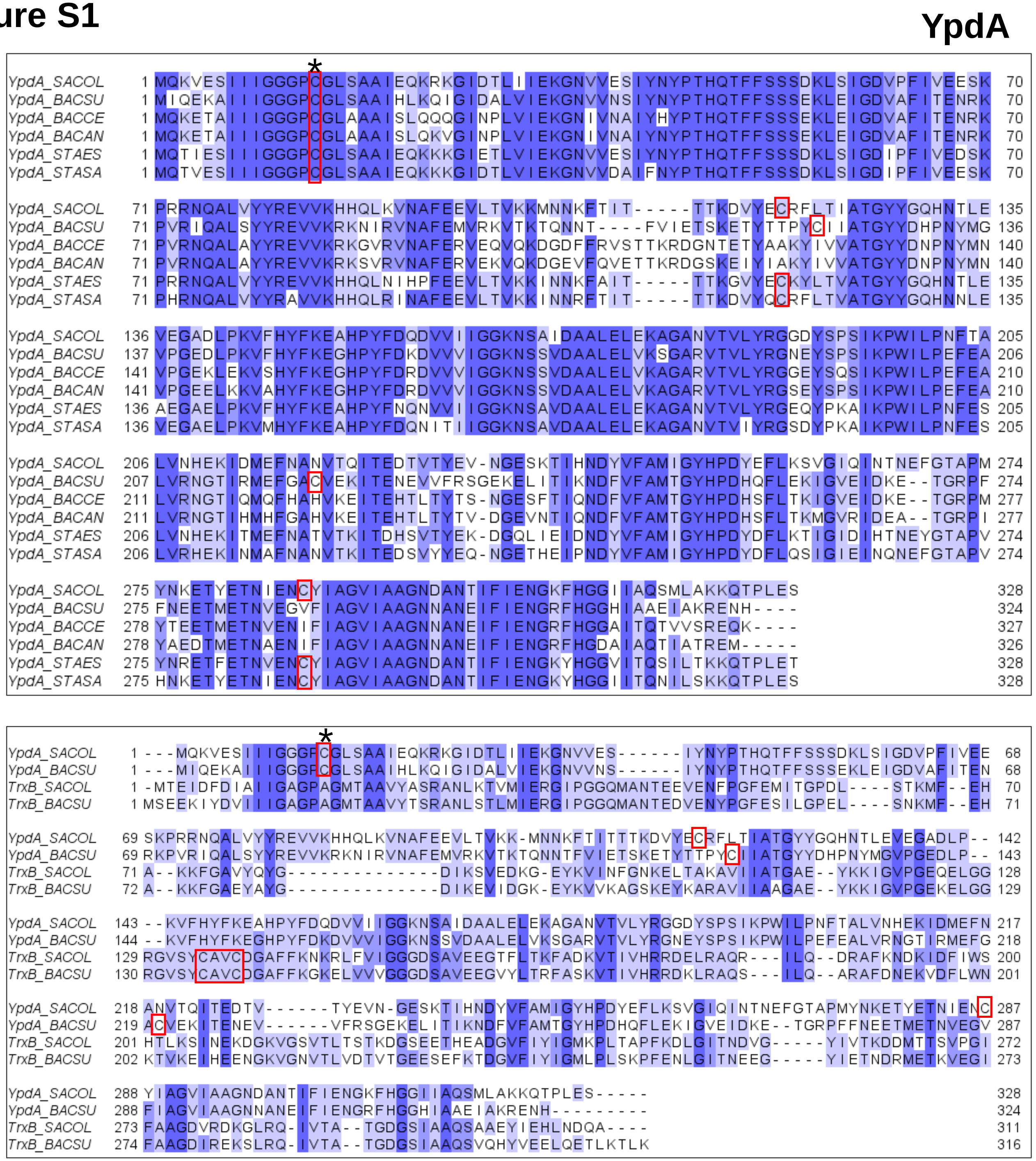


**Fig. S1. ClustalΩ2 protein sequence alignments of BrxA/B and YpdA homologs across firmicutes.** The % identities of the homologs to *S. aureus* BrxA, BrxB and YpdA are given in parenthesis.
**BrxA (SACOL1464)** of *S. aureus* COL was aligned with BrxA homologs of *Staphylococcus epidermidis* (BrxA_STAES, 79.3%), *Staphylococcus saprophyticus* (BrxA_STASA, 73.1%), *B. subtilis* (BrxA_BACSU, 55.1%), *Bacillus cereus* (BrxA_BACCE, 43.2%) and *Bacillus anthracis* (BrxA_BACAN, 43.2%).
**BrxB (SACOL1558**) of *S. aureus* COL was aligned with BrxB homologs of *S. epidermidis* (BrxB_STAES, 87.6%), *S. saprophyticus* (BrxB_STASA, 84.1%), *B. subtilis* (BrxB_BACSU, 68.3%), *B. cereus* (BrxB_BACCE, 68.3%) and *B. anthracis* (BrxB_BACAN, 46.3%).
**YpdA (SACOL1520)** of *S. aureus* COL was aligned with YpdA homologs of *S. saprophyticus* ((YpdA_STASA, 84.1%), *S. epidermidis* (YpdA_STAES, 82.3%), *B. subtilis* (YpdA_BACSU, 62.8%), *B. cereus* (YpdA_BACCE, 61.3%) and *B. anthracis* (YpdA_BACAN, 60.1%). In the lower panel, YpdA of *S. aureus* (SACOL1520) and *B. subtilis* (BSU22950) were aligned with TrxB of *S. aureus* (SACOL0829) and *B. subtilis* (BSU34790). The conserved CGC motif of BrxA/B and the conserved Cys14 residue of YpdA are labeled in red with asterisks (*).

## Figure S2

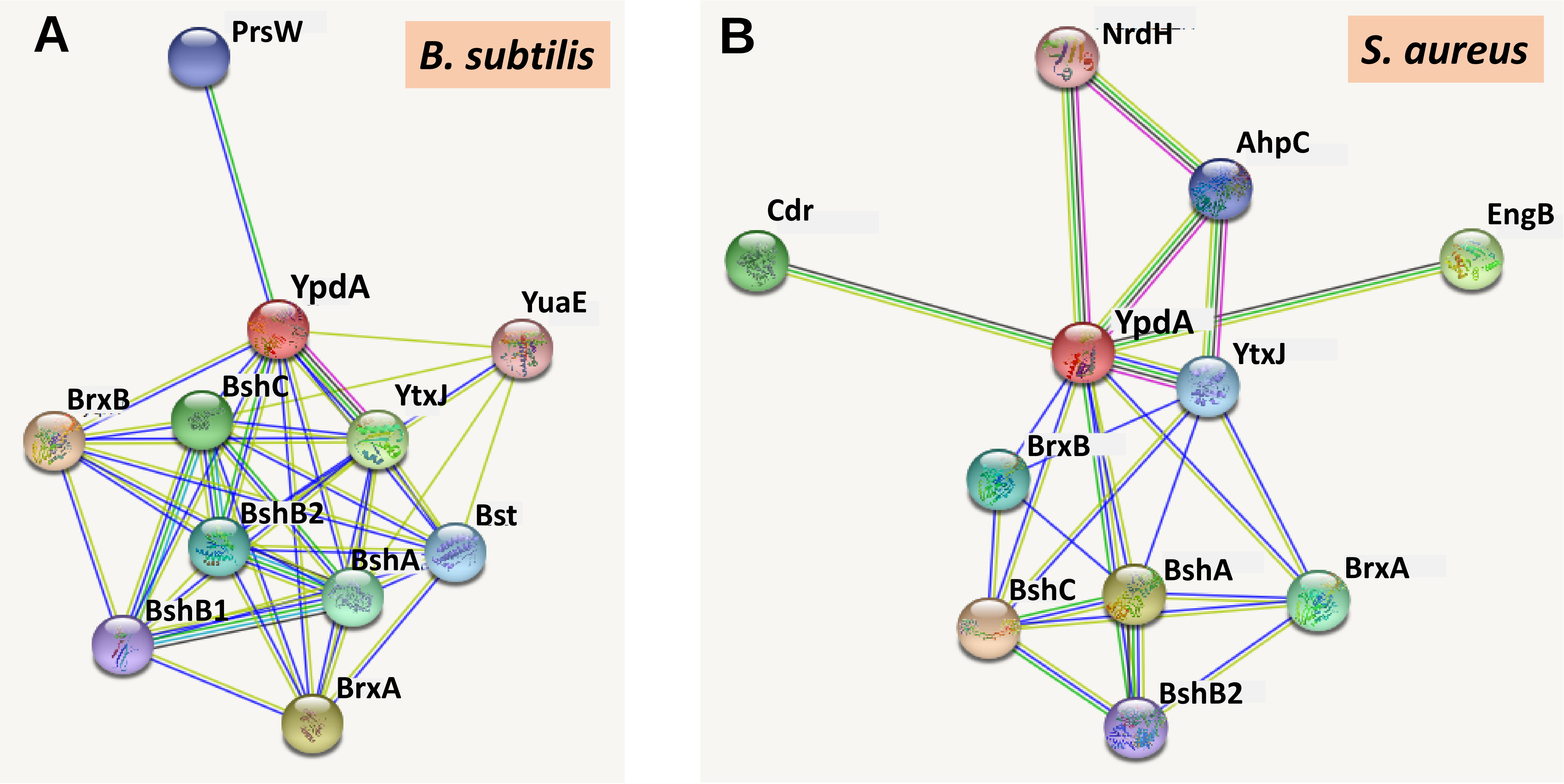


https://string-db.org/cgi/network.pl?taskId=nLwfWwHpDe6G

https://string-db.org/cgi/network.pl?taskId=EAbilN4U7gOB

**Fig. S2. Phylogenomic profiling of YpdA interaction networks with the BSH biosynthesis enzymes (BshA, BshB1/2, BshC) and bacilliredoxins BrxA/B (YphP/YqiW) in *Bacillus subtilis* (A) and *Staphylococcus aureus* NCTC 8325 as revealed by EMBL STRING search (https://string-db.org).** The green and blue lines denote co-localization and co-occurrence of genes in genomes, respectively.

Figure S3

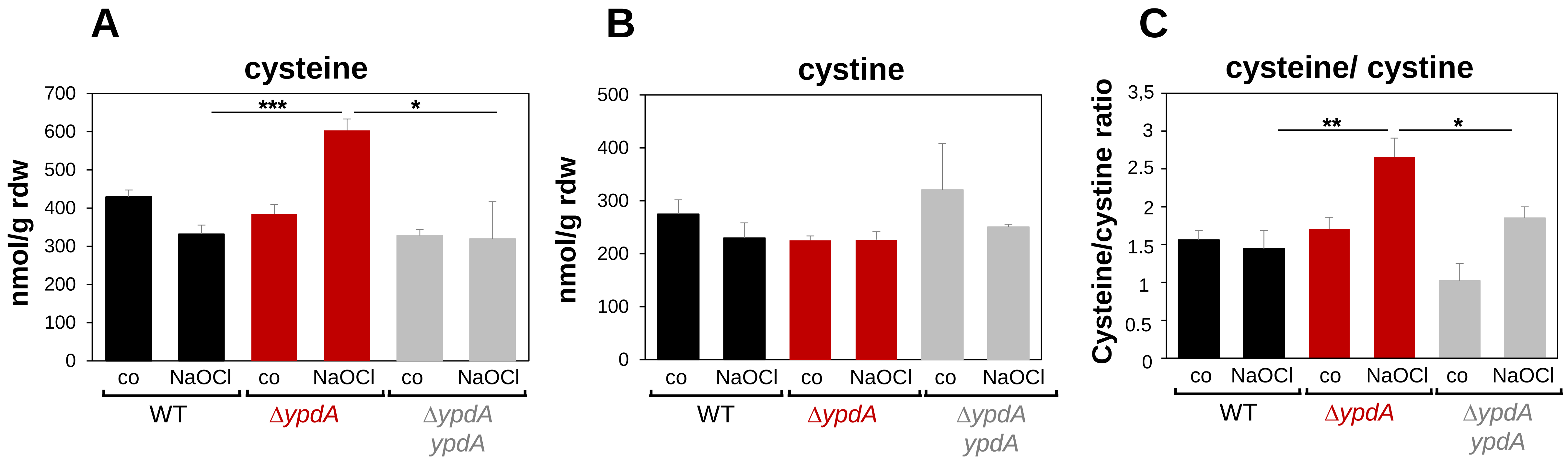


**Fig. S3. Levels of cysteine (A) and cystine (B) and the cysteine/cysteine ratio (C) under control and NaOCl stress in *S. aureus* COL WT, the Δ*ypdA* mutant and *ypdA* complemented strain.** *S. aureus* strains were grown in RPMI and exposed to 2 mM NaOCl stress for 30 min at an $OD_{500}$ of 0.9. mBBr-labeled LMW thiols and disulfides were measured by HPLC thiol metabolomics. Mean values and SD of 3 biological replicates are shown. $^{ns}p > 0.05$; $^{*}p \le 0.05$ $^{**}p \le 0.01$ and $^{***}p \le 0.001$.

## Figure S4

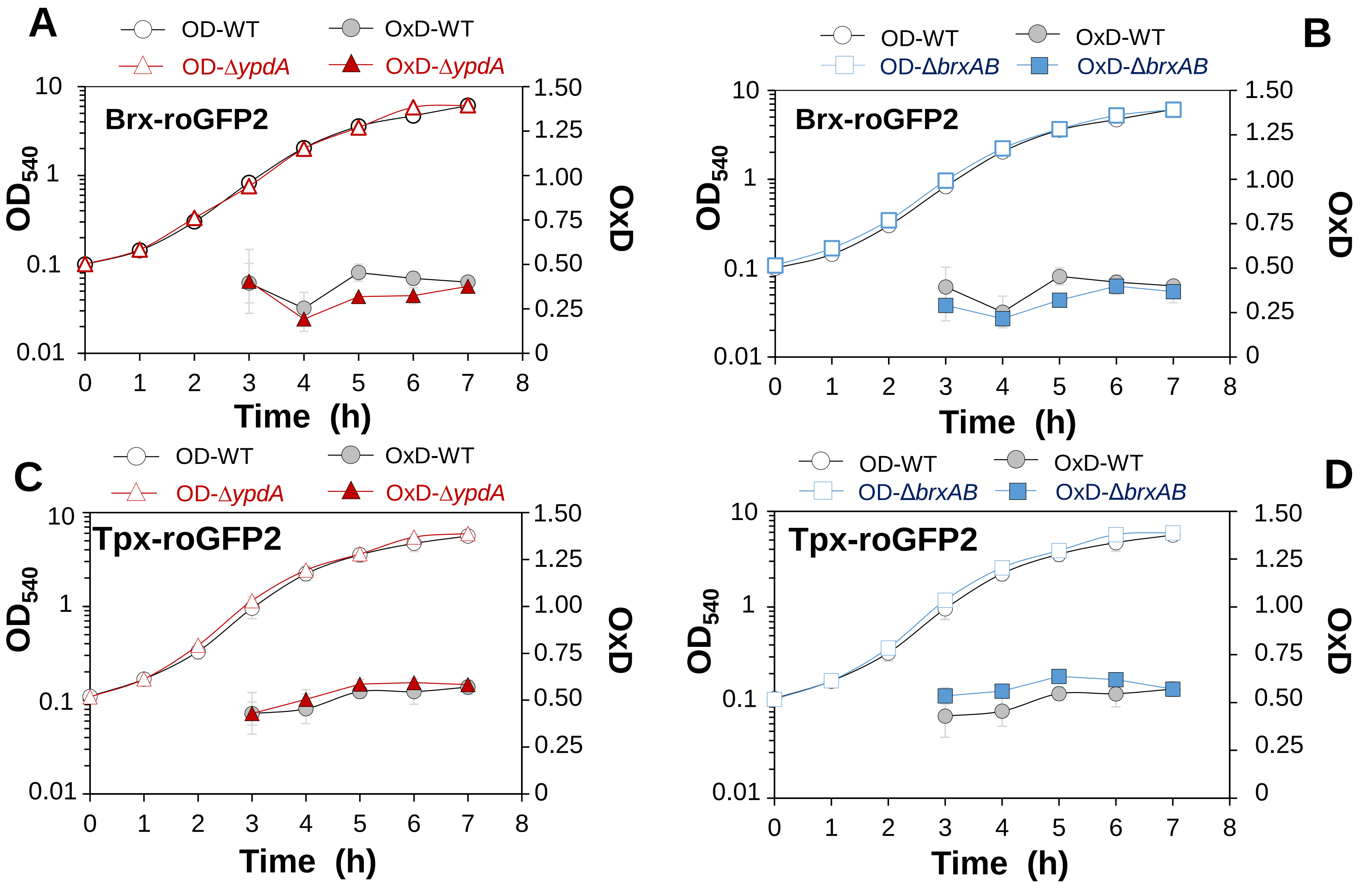


**Fig. S4. The basal level OxD of the Brx-roGFP2 and Tpx-roGFP2 biosensors is not affected in Δ*ypdA* and Δ*brxAB* mutants during the growth.** *S. aureus* COL WT, Δ*ypdA* and Δ*brxAB* mutants expressing Brx-roGFP2 **(A,B)** and Tpx-roGFP2 **(C,D)** were grown in LB medium and the OxD values were determined along the growth curve. Mean values and SD of 3 biological replicates are shown. The corresponding $E_{BSH}$ changes for S4AB were calculated using the Nernst equation in Fig. S5AB.

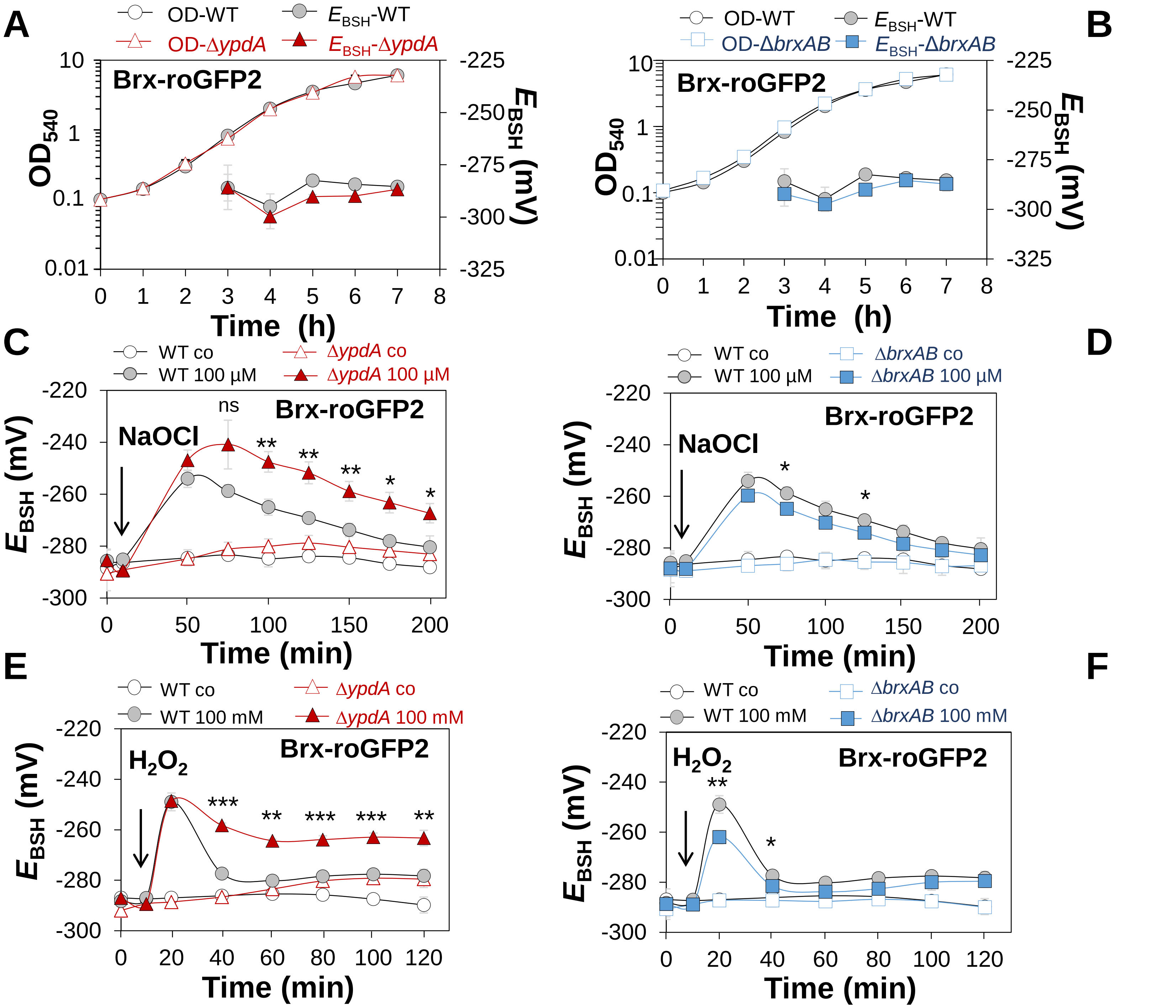


**Fig. S5. YpdA and BrxAB do not affect the basal $E_{BSH}$ level during the growth (A, B), but the Δ*ypdA* mutant is impaired to regenerate the reduced $E_{BSH}$ during recovery from oxidative stress (C, E).**

The $E_{BSH}$ changes were measured in *S. aureus* COL WT, Δ*ypdA* and Δ*brxAB* mutants expressing Brx-roGFP2 along the growth curve in LB **(A, B)** and after exposure to 100 µM NaOCl **(C, D)** or 100 mM $H_2O_2$ stress **(E, F)** in Belitsky minimal medium (BMM). The $E_{BSH}$ values were calculated using the Nernst equation based on the OxD values of Fig. S4AB and Fig. 4A-D.

**Figure S6**

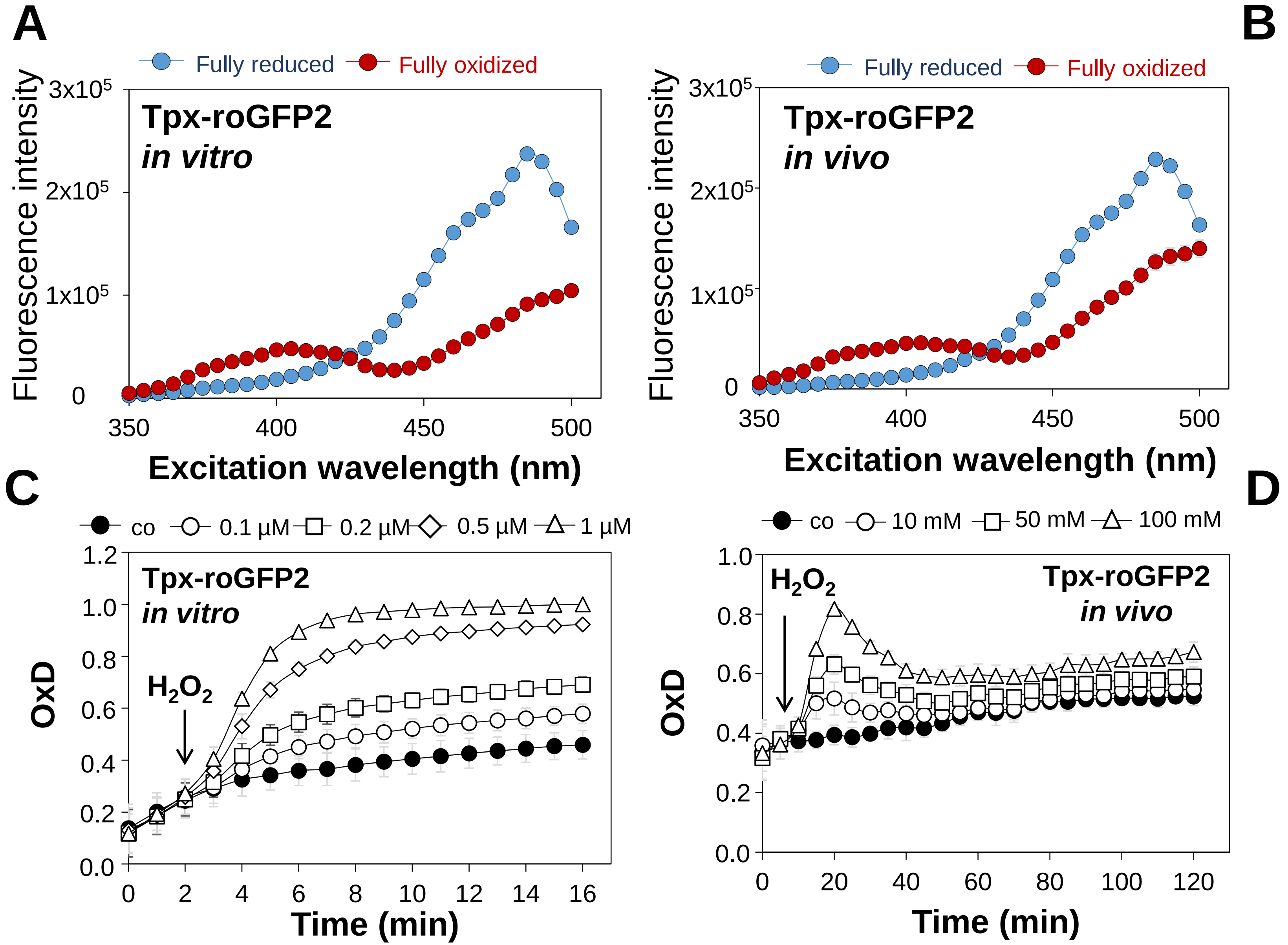


**Fig. S6. Responses of Tpx-roGFP2 *in vitro* (A,C) and *in vivo* inside *S. aureus* COL after exposure to $H_2O_2$ (B,D). (A, B)** The ratiometric Tpx-roGFP2 response in the DTT-treated fully reduced and diamide-treated fully oxidized state *in vitro* **(A)** and inside *S. aureus* COL *in vivo* **(B)**. **(C)** Purified Tpx-roGFP2 (1 µM) responds specifically to low levels $H_2O_2$ (0.1-1 µM $H_2O_2$) *in vitro*. **(D)** Tpx-roGFP2 inside *S. aureus* COL is rapidly and reversible oxidized by sub-lethal 1-100 mM $H_2O_2$ *in vivo*. Mean values and SD of 3-5 replicates are shown.

# Figure S7

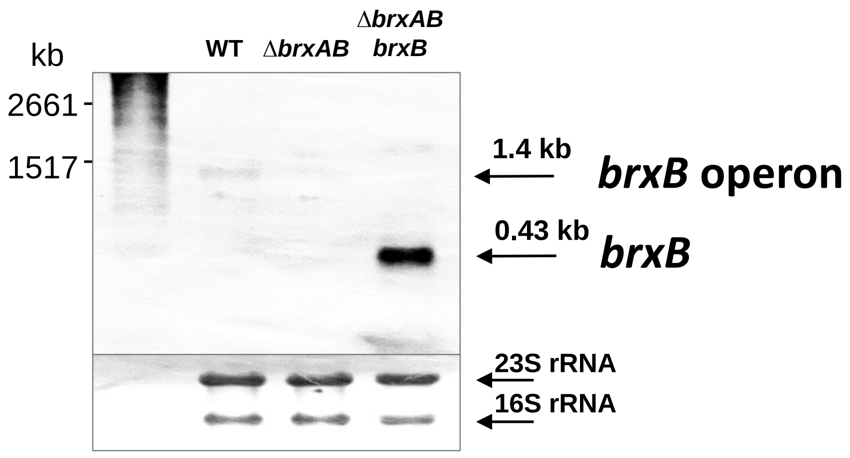


**Fig. S7. Northern blot analysis of *brxB* transcription in the *S. aureus* COL WT, ∆*brxAB* mutant and in the *brxB* complemented strain using a *brxB*-specific RNA probe.** RNA was isolated of *S. aureus* cells grown in LB to an $OD_{540}$ of 2.0 with 1% xylose to induce *brxB* expression in the pRB473-*brxB* complemented ∆*brxAB* mutant strain. *brxB* transcription in the *brxB* complemented strain is shown by the 0.43 kb band on the Northern blot. Methylene blue stained bands for 16S and 23S rRNAs indicate the RNA loading controls below the Northern blot image.

## Figure S8

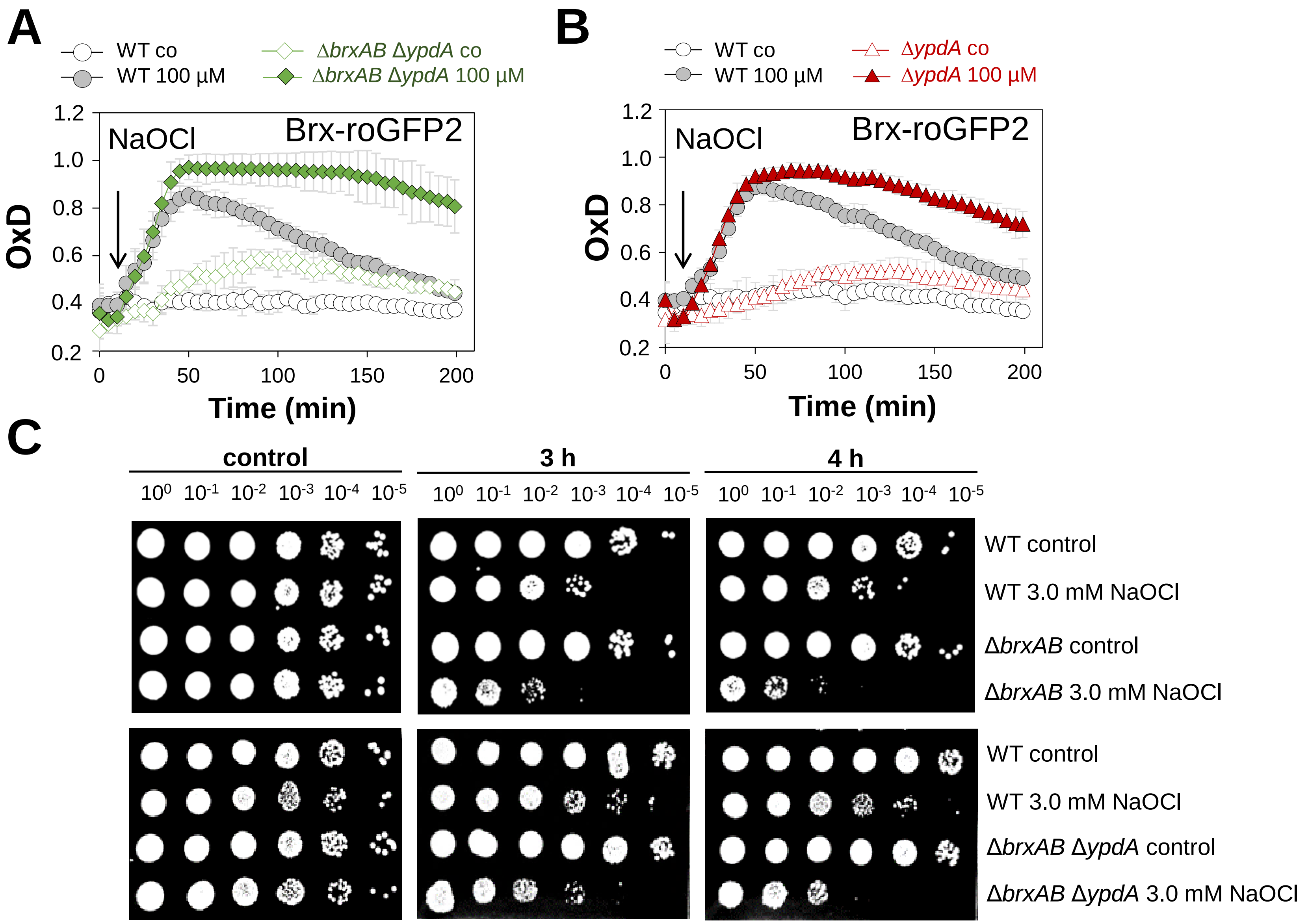


**Fig. S8. Brx-roGFP2 measurements and survival assays indicate that the *S. aureus* ΔbrxAB ΔypdA triple mutant is similar defective to rescue $E_{BSH}$ as the ΔypdA mutant and similar sensitive to NaOCl stress as the ΔbrxAB mutant. (A, B)** Brx-roGFP2 response in the *S. aureus* COL WT, Δ*ypdA* and Δ*brxAB* Δ*ypdA* mutants under 100 µM NaOCl stress**. (C)** Survival assays of the *S. aureus* COL WT, Δ*brxAB* and Δ*brxAB* Δ*ypdA* mutants at 3-4 hours after exposure to 3.0 mM NaOCl.

## Figure S9

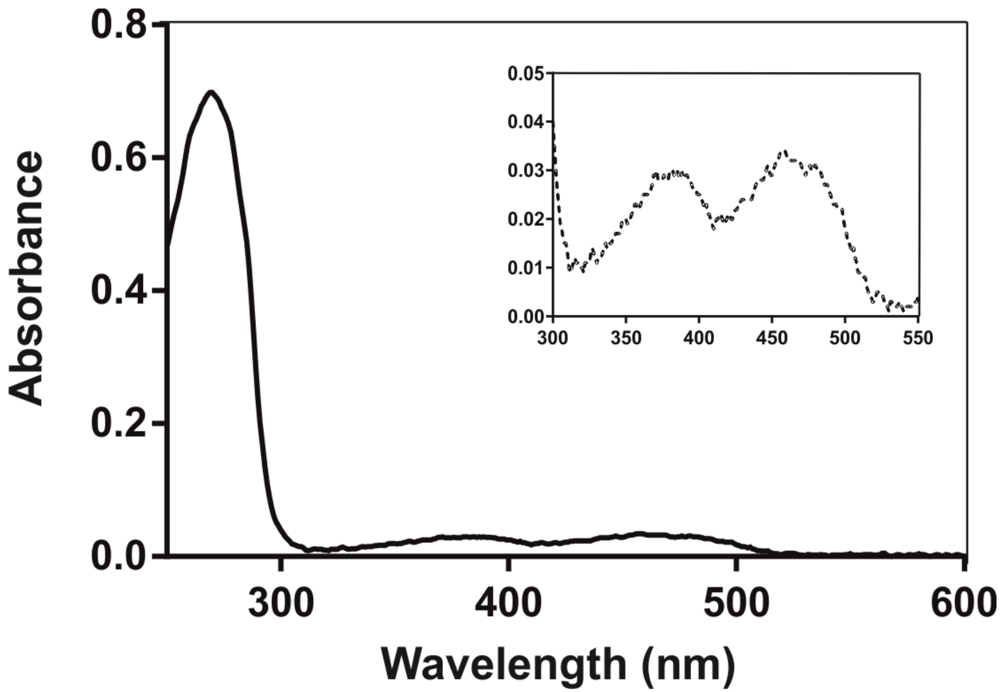


**Fig. S9.** The UV-visible absorption spectrum of purified yellow coloured YpdA protein indicates that YpdA is a flavoprotein containing the FAD co-factor with absorbance peaks at 375 and 450 nm (insert).
