# Supplementary material for: Staphylococcus aureus Uses the Bacilliredoxin (BrxAB)/Bacillithiol Disulfide Reductase (YpdA) Redox Pathway to Defend Against Oxidative Stress Under Infections
Source: Front Microbiol. 2019 Jun 18;10:1355. doi: 10.3389/fmicb.2019.01355 (PMC6591457; doi:10.3389/fmicb.2019.01355)
Supplement: Supplementary file 2 [file Data_Sheet_2.PDF]

Table S1. Bacterial strains

Strain	Description	Reference
Escherichia coli		
DH5α	F-φ80dlacZ Δ(lacZYA-argF) U169 deoRsupE44ΔlacU169 (f80lacZDM15) hsdR17 recA1 endA1 (rk- mk+) supE44gyrA96 thi-1 gyrA69 relA1	[1]
BL21(DE3)*plys*S	F- ompT hsdS gal (rb- mb+) DE3(Sam7 Δnin5 lacUV5-T7 Gen1)	[1]
Staphylococcus aureus		
RN4220	restriction negative strain/MSSA cloning intermediate derived from 8325-4	[2]
COL	Archaic HA-MRSA strain	[3]
COL-Δ*ypdA*	COL *ypdA* deletion mutant	This study
COL-Δ*brxA*	COL *brxA* deletion mutant	This study
COL-Δ*brxAB*	COL *brxAB* double mutant	This study
COL-Δ*brxAB* Δ*ypdA*	COL *brxAB ypdA* triple mutant	This study
COL pRB473		[4]
COL pRB473-*brx-roGFP2*		[4]
COL Δ*ypdA::*pRB473-*brx-roGFP2*		This study
COL Δ*brxAB::*pRB473-*brx-roGFP2*		This study
COL Δ*brxAB* Δ*ypdA::*pRB473-*brx-roGFP2*		This study
COL pRB473-*tpx-roGFP2*		This study
COL Δ*ypdA::*pRB473-*tpx-roGFP2*		This study
COL Δ*brxAB::*pRB473-*tpx-roGFP2*		This study
COL-Δ*ypdA::*pRB473-*ypdA*		This study
COL-Δ*brxAB::*pRB473-*brxA*		This study
COL-Δ*brxAB::*pRB473-*brxB*		This study
Staphylococcus phage 81		[5]

Table S2. Plasmids

Plasmid	Description	Reference
pET11b	*E. coli* expression plasmid	Novagen
pET11b-*ypdA*	pET11b-derivative for overexpression of His-tagged YpdA	This work
pET11b-*ypdAC14A*	pET11b-derivative for overexpression of His-tagged YpdAC14A	This work
pET11b-*brxA*	pET11b-derivative for overexpression of His-tagged BrxA	[6]
pET11b-*gapDH*	pET11b-derivative for overexpression of His-tagged GapDH	[6]
pET11b-*brx-roGFP2*	pET11b-derivative for overexpression of His-tagged Brx-roGFP2	[4]
pET11b-*tpx-roGFP2*	pET11b-derivative for overexpression of His-tagged Tpx-roGFP2	This study
pRB473	pRB373-derivative, *E. coli/ S. aureus* shuttle vector, containing xylose-inducible P_{Xyl} promoter, Amp^r, Cm^r Amp^r, Cm^r	[7, 8]
pRB473-*brx-roGFP2*	pRB473-derivative expressing *brx-roGFP2* under P_{Xyl}	[4]
pRB473-*tpx-roGFP2*	pRB473-derivative expressing *tpx-roGFP2* under P_{Xyl}	This study
pRB473-*ypdA*	pRB473-derivative expressing *ypdA* under P_{Xyl}	This study
pRB473-*brxA*	pRB473-derivative expressing *brxA* under P_{Xyl}	This study
pRB473-*brxB*	pRB473-derivative expressing *brxB* under P_{Xyl}	This study

Table S3. Oligonucleotide primers

Primer name	Sequence (5' to 3')
pET-tpx-for-NheI	CTAGCTAGCATGACTGAAATAACATTCAAAGG
pET-tpx-rev-SpeI	GCGACTAGTAATATTTTTGTATGCAGCTAAAGC
pET-ypdA-for-NdeI	GGAATTCCATATGCAAAAAGTTGAAAGTATCATA
pET-ypdAC14A-for-NdeI	GGAATTCCATATGCAAAAAGTTGAAAGTATCATAATTGGTGGAGGGCCA**GCG**GGATT
pET-ypdA-rev-BamHI	CGCGGATCCTTAGTGATGGTGATGGTGATGTGATTCTAAGGGCGTTTGTTTC
pRB-tpx-roGFP2-for-BamHI	CGCGGATCCTTAGTGATGGTGATGGTGATGTGATTCTAAGGGCGTTTGTTTC
pRB-tpx-roGFP2-rev-SacI	CGCGAGCTCTTACTTGTACAGCTCGTCCATGC
pMAD-ypdA-for-BglII	CGCAGATCTGACATACAGTGAATGGTCAAG
pMAD-ypdA-f1-rev	GTTATTTAGTACATAGACCTTTATTTCATTGTTTCGGCCTCCTTTAATC
pMAD-ypdA-f2-for	GATTAAAGGAGGCCGAAACAATGAAATAAAGGTCTATGTACTAAATAAC
pMAD-ypdA-rev-SalI	CCAGTCGACTGTATTGACAAAGGATCGTGTG
pMAD-brxA-for-BglII	CGCAGATCTCGATCATTTCGTGTATTTCCTA
pMAD-brxA-f1-rev	TAAATATGAATGCATATGATGCTCCTTTGACGAAAATTGTAAATAGT
pMAD-brxA-f2-for	ACTATTTACAATTTTCGTCAAAGGAGCATCATATGCATTCATATTTA
pMAD-brxA-rev-SalI	CCAGTCGACTGGAAGACTCGATTACGAATG
pMAD-brxB-for-BglII	CGCAGATCTCAATCGCAATGGTATCTTCATA
pMAD-brxB-f1-rev	GGATAGGTGATTGAACTTATGGATTGTGAAGAAAGATAAGAGGC
pMAD-brxB-f2-for	GCCTCTTATCTTTCTTCACAATCCATAAGTTCAATCACCTATCC
pMAD-brxB-rev-SalI	CCAGTCGACTGATATGATTGCAATTCGTAAC
pRB-ypdA-for-BamHI	TAGGGATCCTTAAAGGAGGCCGAAACAATGCAAAAAGTTGAAAGTATCA
pRB-ypdA-rev-KpnI	CTCGGTACCTTATGATTCTAAGGGCGTTTG
pRB-brxA-for-BamHI	TAGGGATCCTAATTGGAGGAATTAAATATGAAT
pRB-brxA-rev-KpnI	CTCGGTACCCTATTTACAATTTTCGTCAAAGG
pRB-brxB-for-BamHI	TAGGGATCCGGATAGGTGATTGAACTTATGG
pRB-brxB-rev-KpnI	CTCGGTACCTTATCTTTCTTCACAATATTTATTG
ypdA-NB-for	GGGCCATGCGGATTAAGTG
ypdA-NB-rev	CTAATACGACTCACTATAGGGAGACGTTCCCTGCAGCAATTACA
brxA-NB-for	GCTTATATGAAAGAAATTGCGC
brxA-NB-rev	CTAATACGACTCACTATAGGGAGAAATTTTCGTCAAAGGCATCCTT
brxB-NB-for	TTATACATGAACGGTGTTGTAG
brxB-NB-rev	CTAATACGACTCACTATAGGGAGAATTACGTTCATCACATCATGAC

Restriction sites are underlined.

Table S4. The basal BSH redox potential (E_{BSH}) of *S aureus* COL wild type (WT), Δ*ypdA* and Δ*brxAB* mutants at different time points during the growth curve in LB medium

Time (h)	WT		Δ*ypdA*		Δ*brxAB*	
	OD_{540}	E_{BSH} (mV)	OD_{540}	E_{BSH} (mV)	OD_{540}	E_{BSH} (mV)
**3**	0.83	-285.93 ± 6.36	0.76	-292.36 ± 6.00	0.97	-285.79 ± 10.63
**4**	2.04	-294.90 ± 6.04	1.99	-297.44 ± 3.58	2.22	-299.73 ± 5.82
**5**	3.57	-282.51 ± 2.60	3.46	-290.10 ± 0.94	3.66	-290.16 ± 0.98
**6**	4.69	-284.26 ± 2.14	5.83	-285.51 ± 2.62	5.22	-289.88 ± 2.71
**7**	6.10	-285.45 ± 0.93	6.13	-288.31 ± 3.53	6.07	-286.75 ± 0.31

Table S5. Effect of 100 µM NaOCl on E_{BSH} changes in *S aureus* COL WT, Δ*ypdA* and Δ*brxAB* mutants

Time (min)	E_{BSH} (mV)		
	WT	Δ*ypdA*	Δ*brxAB*
**0**	-285.73 ± 4.57	-285.48 ± 3.95	-287.92 ± 5.60
**10**	-285.18 ± 1.22	-289.49 ± 0.27	-288.12 ± 2.95
**50**	-254.04 ± 3.33	-247.00 ± 3.96	-258.69 ± 2.47
**75**	-258.80 ± 2.40	-240.87 ± 9.39	-264.83 ± 1.03
**100**	-265.00 ± 3.09	-247.58 ± 3.92	-270.21 ± 0.55
**125**	-269.22 ± 1.91	-251.82 ± 4.21	-274.14 ± 1.10
**150**	-273.76 ± 2.56	-258.88 ± 3.81	-278.43 ± 1.26
**175**	-278.06 ± 1.91	-263.24 ± 3.95	-280.87 ± 1.35
**200**	-280.43 ± 4.33	-267.35 ± 3.74	-282.80 ± 1.36

Table S6: Effect of 100 mM H_2O_2 on E_{BSH} changes in *S aureus* COL WT, Δ*ypdA* and Δ*brxAB* mutants

Time (min)	E_{BSH} (mV)		
	WT	Δ*ypdA*	Δ*brxAB*
**0**	-288.25 ± 1.72	-287.04 ± 2.04	-288.77 ± 6.09
**10**	-287.00 ± 1.78	-289.33 ± 1.13	-288.96 ± 1.38
**20**	-248.94 ± 3.54	-248.62 ± 0.98	-261.96 ± 2.63
**40**	-277.32 ± 1.91	-258.26 ± 0.93	-281.54 ± 1.36
**60**	-280.16 ± 2.00	-264.35 ± 0.63	-283.82 ± 1.36
**80**	-278.40 ± 1.69	-263.89 ± 1.50	-282.60 ± 3.31
**100**	-277.58 ± 1.93	-262.93 ± 1.60	-280.03 ± 3.15
**120**	-278.25 ± 2.64	-263.27 ± 3.09	-279.60 ± 2.38

Supplementary References

[1] F.W. Studier, B.A. Moffatt, Use of bacteriophage-T7 RNA-polymerase to direct selective high-level expression of cloned genes, J Mol Biol 189(1) (1986) 113-130.

[2] B.N. Kreiswirth, S. Lofdahl, M.J. Betley, M. O'Reilly, P.M. Schlievert, M.S. Bergdoll, R.P. Novick, The toxic shock syndrome exotoxin structural gene is not detectably transmitted by a prophage, Nature 305(5936) (1983) 709-12.

[3] W.M. Shafer, J.J. Iandolo, Genetics of staphylococcal enterotoxin B in methicillin-resistant isolates of *Staphylococcus aureus*, Infect Immun 25(3) (1979) 902-11.

[4] V.V. Loi, M. Harms, M. Müller, N.T.T. Huyen, C.J. Hamilton, F. Hochgräfe, J. Pane-Farre, H. Antelmann, Real-time imaging of the bacillithiol redox potential in the human pathogen *Staphylococcus aureus* using a genetically encoded bacilliredoxin-fused redox biosensor, Antioxid Redox Signal 26(15) (2017) 835-848.

[5] E.D. Rosenblum, S. Tyrone, Serology, density, and morphology of staphylococcal phages, J Bacteriol 88 (1964) 1737-42.

[6] M. Imber, N.T.T. Huyen, A.J. Pietrzyk-Brzezinska, V.V. Loi, M. Hillion, J. Bernhardt, L. Thärichen, K. Kolsek, M. Saleh, C.J. Hamilton, L. Adrian, F. Gräter, M.C. Wahl, H. Antelmann, Protein S-Bacillithiolation functions in thiol protection and redox regulation of the glyceraldehyde-3-phosphate dehydrogenase Gap in *Staphylococcus aureus* under hypochlorite stress, Antioxid Redox Signal 28(6) (2018) 410-430.

[7] D.C. Pöther, P. Gierok, M. Harms, J. Mostertz, F. Hochgräfe, H. Antelmann, C.J. Hamilton, I. Borovok, M. Lalk, Y. Aharonowitz, M. Hecker, Distribution and infection-related functions of bacillithiol in *Staphylococcus aureus*, Int J Med Microbiol 303(3) (2013) 114-23.

[8] R. Brückner, E. Wagner, F. Götz, Characterization of a sucrase gene from *Staphylococcus xylosus*, J Bacteriol 175(3) (1993) 851-7.
